# Formulate Adaptive Biphasic Scaffold via Sequential Protein‐Instructed Peptide Co‐Assembly

**DOI:** 10.1002/advs.202401478

**Published:** 2024-05-24

**Authors:** Yazhou Chen, Qizheng Zhang, Shenyu Yang, Guanying Li, Chaochen Shi, Xunwu Hu, Shunsuke Asahina, Natsuko Asano, Ye Zhang

**Affiliations:** ^1^ Henan Institute of Advanced Technology Zhengzhou University Zhengzhou Henan 450003 China; ^2^ Active Soft Matter Group Songshan Lake Materials Laboratory Dongguan Guangdong 523808 China; ^3^ Medical 3D Printing Center The First Affiliated Hospital of Zhengzhou University Zhengzhou University Zhengzhou Henan 450052 China; ^4^ Department of Biophysics School of Basic Medical Sciences Health Science Center Xi'an Jiaotong University Xi'an Shaanxi 71006 China; ^5^ SM Application Planning Group JEOL Ltd. Akishima Tokyo 196‐8588 Japan; ^6^ Institute of Multidisciplinary Research for Advanced Materials (IMRAM) Tohoku University Sendai 980‐8572 Japan

**Keywords:** adaptive scaffold, biphasic scaffold, dual‐targeting peptide, peptide‐co‐assembly, protein‐instructed peptide assembly

## Abstract

To ensure compositional consistency while mitigating potential immunogenicity for stem cell therapy, synthetic scaffolds have emerged as compelling alternatives to native extracellular matrix (ECM). Substantial progress has been made in emulating specific natural traits featuring consistent chemical compositions and physical structures. However, recapitulating the dynamic responsiveness of the native ECM involving chemical transitions and physical remodeling during differentiation, remains a challenging endeavor. Here, the creation of adaptive scaffolds is demonstrated through sequential protein‐instructed molecular assembly, utilizing stage‐specific proteins, and incorporating in situ assembly technique. The procedure is commenced by introducing a dual‐targeting peptide at the onset of stem cell differentiation. In response to highly expressed integrins and heparan sulfate proteoglycans (HSPGs) on human mesenchymal stem cell (hMSC), the peptides assembled in situ, creating customized extracellular scaffolds that adhered to hMSCs promoting osteoblast differentiation. As the expression of alkaline phosphatase (ALP) and collagen (COL‐1) increased in osteoblasts, an additional peptide is introduced that interacts with ALP, initiating peptide assembly and facilitating calcium phosphate (CaP) deposition. The growth and entanglement of peptide assemblies with collagen fibers efficiently incorporated CaP into the network resulting in an adaptive biphasic scaffold that enhanced healing of bone injuries.

## Graphical abstract

Utilizing a sequential protein‐instructed molecular assembly approach, adaptive scaffolds are developed that emulate the dynamic responsiveness of the native extracellular matrix (ECM). By incorporating peptides interacting with stage‐specific proteins during osteogenesis, the scaffolds demonstrate remarkable adaptability to the everchanging cellular microenvironment enhancing bone injury healing.

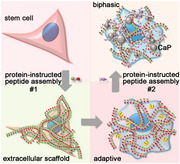

## 1 Introduction

Stem‐cell‐based tissue engineering is an ever‐evolving field with immense potential for tissue repair and regeneration.^[^ ^1^ ^]^ The progress of engineering strategies hinges on the effective and safe orchestration of cell differentiation. In nature, stem cell fate is directly affected by the interaction of the cells with their surrounding extracellular matrix (ECM),^[^ ^2^ ^]^ whereby mechanical cues and intrinsic biochemical factors act in concert to give rise to a series of spatially and temporally coordinated events that regulate cell differentiation and function.^[^ ^3^ ^]^ Over the past four decades, nature‐derived ECM materials, rich in a diverse array of proteins and signaling molecules, have provided a biologically authentic microenvironment for numerous cell culture and applications. However, their ill‐defined and variable composition, coupled with limited responsiveness to physical or biochemical manipulation, posing challenges for precise tuning to elicit desired cell behaviors and achieve specific biological outcomes. As anticipated, the design of synthetic scaffolds tailored to faithfully replicate the morphology, mechanics, and dynamics of the specific ECM requirements providing the tightly governed spatiotemporal cues during stem cell differentiation holds great promise for advancing tissue reconstruction.^[^ ^4^ ^]^

Over the past two decades, substantial progress has been made in the utilization of synthetic polymers as extracellular scaffolds. Notably, key parameters such as scaffold stiffness, degradability, as well as the incorporation of tethered cell adhesion peptides and growth factors, have undergone systematic adjustments in terms of composition, molecular weight, crosslinker type, crosslink density, and polymerization methods.^[^ ^5^ ^]^ These tailored polymeric materials provided relatively static microenvironment to foster stem‐cell differentiation by aligning with a specific cellular process. However, the differentiation of stem cells entails a complex interplay of multiple cellular processes, necessitating continuous modification of ECM to accommodate these dynamic changes.^[^ ^3^ ^]^ Recently, mechanically dynamic polymers^[^ ^6^ ^]^ with tunable crosslink density via light, chemical composition, sound waves, and reversible hydrogen bonding were developed to emulate reshaping process,^[^ ^7^ ^]^ however, achieving ongoing modification physically and chemically to adapt to a series of diverse cellular processes remains a formidable challenge. Rather than predefining initial parameters conductive to cell function, relying on cell‐mediated processes to dynamically optimize material properties may hold the key to construct scaffolds that adapt to the intricacies of stem cell differentiation.

**Figure 1:**
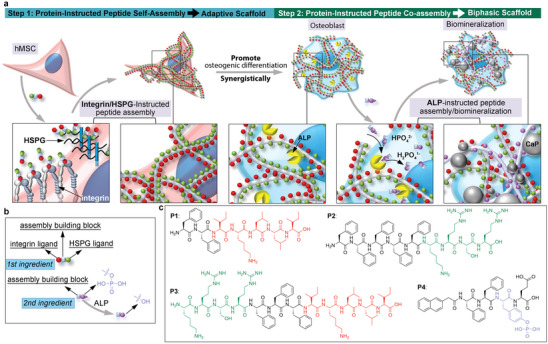
Stepwise construction of adaptive biphasic scaffold for osteogenic differentiation of hMSCs. a) Schematic illustration of adaptive biphasic scaffold assembled by hMSC encompassing osteogenic differentiation process. b) Schematic illustration of the design of two peptides for sequential protein‐instructed peptide assembly. c) Chemical structures of synthetic peptides applied in this research.

Chen lab and Ariga Lab have been at the forefront of this endeavor. Chen lab revealed the importance of cell‐mediated ECM rearrangement for human mesenchymal stem cells (hMSCs) proliferation.^[^ ^8^ ^]^ Following that, Ariga lab pioneered the development of adaptive scaffolds for hMSCs differentiation.^[^ ^9^ ^]^ Their groundbreaking efforts seamlessly integrate stem cell mediation into scaffold creation, aligning extracellular protein nanofibers through the innovative concept of liquid‐liquid interfacial jamming facilitated by hMSCs‐exerted traction force.^[^ ^10^ ^]^ This approach, which embodies the principles of nanoarchitectonics, has yielded remarkable success, particularly in the promotion and induction of neuronal differentiation. While liquid‐liquid interface methods hold promise for advancing our understanding of stem cell‐matrix interactions, their practical application in biomedicine faces limitations. In response, we propose a well‐conceived design for a cell‐assembled biphasic scaffold. It integrates reciprocal interactions between hMSCs and synthetic scaffolds throughout osteogenic differentiation into the construction and remodeling process through a sequential protein‐biomarker‐instructed peptide assembly (PBIPA) approach (**Figure** **1**a).

Enzyme‐instructed self‐assembly (EISA), a specific form of PBIPA triggered by enzyme catalytic reactions, has garnered significant attention in cancer research due to its ability to form pericellular nanonets on cancer cells.^[^ ^11^ ^]^ Utilizing specific alkaline phosphatase localized on the plasma membrane has paved the way for constructing therapeutic extracellular scaffolds that suppress cancer cell proliferation. Additionally, our recent studies introduced another form of PBIPA based on the specificity of protein‐ligand binding. By incorporating ligands of cancer biomarker proteins integrins and heparan sulfate proteoglycans (HSPGs) into the assembly building blocks (Figure 1b),^[^ ^12^ ^]^ we developed assembling ligands **P1** and **P2** (Figure 1c).^[^ ^13^ ^]^ Through PBIPA, both ligands self‐assemble forming extracellular scaffolds selectively adhere to the apical membrane of cancer cells. These soft and flexible scaffolds effectively inhibit cancer cell migration without inducing cytotoxicity. Building on this knowledge, our objective is to utilize PBIPA to construct adaptive scaffolds, emphasizing the continuous reciprocal interactions between stem cells and peptide assemblies. These interactions are spatiotemporally orchestrated by protein biomarkers corresponding to sequential stages of osteogenic differentiation (Figure 1a,b).

## 2 Results and Discussion

Integrins and proteoglycans (PGs) serve as primary ECM adhesion receptors contributing jointly to signaling events before and during osteogenic differentiation. Integrin ligands are utilized as pendant or crosslinkers in polymeric scaffolds to promote osteogenic differentiation,^[^ ^14^ ^]^ while tethering HSPG ligands in synthetic scaffolds remains unexplored despite HSPGs being demonstrated to mediate osteoblast adhesion.^[^ ^15^ ^]^ To trigger the synergistic contribution of both integrin and HSPG in orchestrating osteogenic differentiation,^[^ ^16^ ^]^ we developed a dual‐targeting peptide assembly (Figure 1b). Laminin‐derived peptide IKLLI, binding with both integrin  α_3_ subunit and integrin β_1_ subunit that are up‐regulated during osteogenic differentiation,^[^ ^17^ ^]^ and the “Cardin‐Weintraub” sequence KRSR, binding with HSPG, are selected to be bridged with three _L_‐phenylalanine repeats, resulting in **P3** for the first PBIPA, integrin/HSPG‐instructed peptide assembly (Figure 1c; Figures S1–S3, Supporting Information). Following the osteogenic differentiation of hMSCs into osteoblast, alkaline phosphatase (ALP) expression is increased, facilitating ECM mineralization^[^ ^18^ ^]^ with calcium phosphate (CaP) infiltrating collagen fibrils, which is critical for bone regeneration.^[^ ^19^ ^]^ To allow hMSCs mediate biphasic scaffold construction,^[^ ^20^ ^]^ here we introduced a phosphorylated peptide **P4** that shares the same assembling building block _L_‐phenylalanine repeats (Figure 1c; Figures S4–S7, Supporting Information) to initiate the second PBIPA,ALP‐catalyzed peptide assembly and in situ calcium deposition.^[^ ^21^ ^]^

Under the instruction of integrin and HSPG on cancer cell membrane, peptides **P1** and **P2** autonomously assemble into extracellular scaffolds, a phenomenon thoroughly characterized in our prior investigation.^[^ ^13b,c^ ^]^ As a structural combination of both **P1** and **P2**, peptide **P3** demonstrates enhanced hydrophilicity compared to **P1** and undergoes self‐assembly at a lower concentration than **P2**. Initially, **P3** forms nanofragments in water at 25 µM, which transition into nanofibers with increased concentration beyond 100 µM (Figure S8a, Supporting Information). While **P3** self‐assembles at low concentrations, it exhibits limited hydrogel formation until reaching super high concentrations beyond 30 mM (Figure S8b, Supporting Information), without the typical swelling behavior observed in most polymeric hydrogels. The digestion assay suggests that self‐assembly slightly reduces the degradation rate of the peptides (Figure S8c, Supporting Information). Upon mixing with heparin, a polysaccharide sharing common structural features with the side chain of HSPG, **P3** orchestrates the formation of dense fibrous networks at low concentrations, showcasing HSPG's instructive role in promoting **P3** assembly (Figure S8d, Supporting Information). Upon ALP treatment, peptide **P4** undergoes hydrolysis, transforming into a hydrophobic derivative that self‐assembles forming nanofibers (Figure S8e, Supporting Information). In the course of this ALP‐instructed peptide **P4** assembly, the presence of calcium ions triggers in situ calcium deposition, embedding CaP within the nanofibrous network (Figure S8f, Supporting Information).

The co‐assembly of **P3** and **P4** was scrutinized and verified through electron microscopy imaging (Figure S9a, Supporting Information) and circular dichroism (CD) analysis (Figure S9b, Supporting Information). Both TEM and SEM images illustrate a morphological transition from thicker and straight nanofibers to thinner and bending nanofibers when comparing the self‐assembly of **P3** with the co‐assembly of **P3** and **P4**. The self‐assembly of **P3** exhibits distinct CD signals compared to the self‐assembly of **P1** or **P2**, both of which form β‐sheet structures. This difference may be attribute to the positioning of assembling building blocks, at one side of **P1** or **P2** but in the center of **P3**. The mixture of **P3** and **P4** shows different CD signals from the simple sum of CD signals of **P3** and **P4**, confirming co‐assembly. Furthermore, the simultaneous introduction of ALP and calcium ions to the co‐assemblies induced calcium deposition that permeated into a more compact fibrous network, ultimately culminating in the formation of a biphasic scaffold (Figure S9a,c,d, Supporting Information).

**Figure 2:**
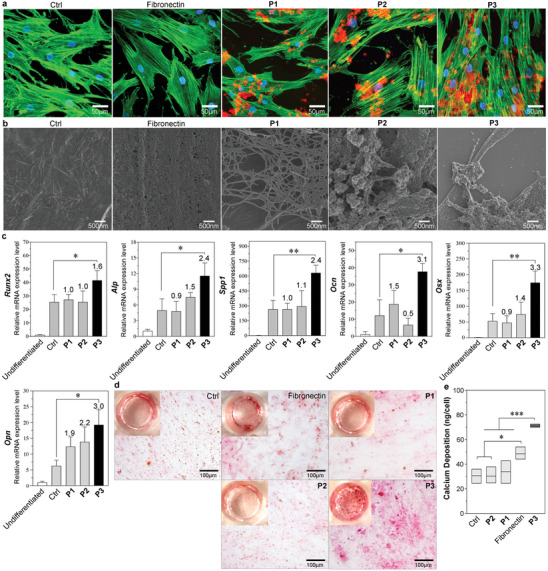
Dual‐targeting assembly peptide **P3** self‐assembles on the apical membrane of hMSCs promoting osteogenic differentiation. a) Fluorescent images of hMSCs cultured with 1xPBS (Ctrl), on fibronectin‐coated surface, with **P1**, **P2**, and **P3** at a concentration of 200 µM for 3 days, co‐stained with Congo Red, ActinGreen and DAPI. b) SEM images of hMSCs surfaces correlated to panel a. c) Relative quantification of gene expression in hMSCs upon the treatment of **P1**, **P2** or **P3** in osteogenic medium for 21 days normalized to undifferentiated state (mean ± SD, *n* = 3). *p < 0.1, ** *p* < 0.01. The numbers upon the columns represent the fold change of gene expression relative to the control condition (Ctrl). d) Optical images of Alizarin red stained hMSCs upon the treatment of 1xPBS, peptides **P1**–**P3** at a concentration of 200 µm, and cultured on fibronectin coated surface for 21‐day osteogenic differentiation. e) Quantified calcium deposition in hMSCs corelated to panel d (mean ± SD, *n* = 3). *p < 0.1, **p < 0.01, ***p < 0.001.

Prior to applying the peptides for hMSC‐mediated construction of extracellular scaffold, we conducted preliminary biocompatibility tests. Additionally, to verify the function of each building block of **P3**, we synthesized several peptides: **P5** (abbreviated as NapFF) (Figures S10 and S11, Supporting Information), which serves as a building block for molecular self‐assembly but lacks binding affinity with integrin or HSPG; **P6** (IKLLI) (Figure S12, Supporting Information), which acts as the ligand for integrin α_3_β_1_ but does not possess self‐assembling ability; and **P7** (KRSR) (Figure S13, Supporting Information), a hydrophilic peptide that binds with HSPG. Notably, the assembling ligands **P1**, **P2**, and **P3**, as well as the neutral peptide **P5**, and the mixture of **P6** and **P7** at a 1:1 ratio representing the integrin/HSPG binding building blocks of **P3** with no self‐assembling ability, exhibited good biocompatibility with hMSCs (Figures S14–S16, Supporting Information). Treatment with peptide **P1**, **P2**, or **P3** at a concentration of 200 µm, significantly lower than the gelation concentration, led to the formation of extracellular scaffolds adhering to the apical membrane of hMSCs (**Figure** **2**a,b; Figure S17, Supporting Information). In specific, **P1** self‐assembled into a fibrous scaffold, while both **P2** and **P3** self‐assembled into aggregation scaffolds with fewer coarse aggregates in **P3** assemblies. At concentrations exceeding 200 µM, **P3** treatment triggered an elevation of ALP activity and elongation of hMSCs (Figure S18, Supporting Information). Comparing to other peptides, **P3** treatment induced the highest ALP activity during osteogenesis, surpassing the classical fibronectin coating assay^[^ ^22^ ^]^ that promotes osteogenic differentiation through adhesion regulation (Figure S19, Supporting Information). Consistently, **P3** treatment markedly up‐regulated bone markers, including gene expressions of *Runx2*, *Alp*, *Spp1*, *Ocn*, *Osx*, and *Opn*, as well as protein expression of RUNX2, ALP, and OSX, compared to control and mono‐targeting assembling peptides **P1** and **P2** (Figure 2c; Figure S20, Supporting Information), suggesting a robust promotion of osteogenesis through the synergistic contributions of dual targeting design. In the presence of the extracellular scaffold assembled by **P3**, hMSCs deposited significantly more calcium, as evidenced by alizarin red stain (Figure 2d).

**Figure 3:**
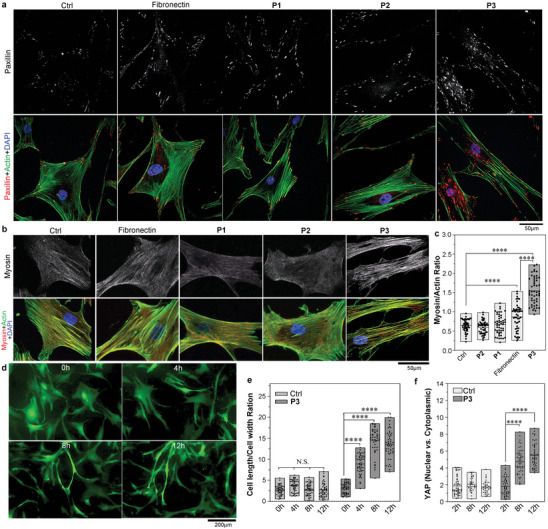
Self‐assembly of **P3** induces mechanotransduction in hMSCs promoting osteogenic differentiation. a) Immunofluorescence^[^ ^28^ ^]^ staining of paxillin on hMSCs upon the treatment of 1xPBS (Ctrl), **P1**–**P3** (200 µm), or cultured on fibronectin coated surface (Fibronectin) for 3 days co‐stained with ActinGreen and DAPI. (b) IF staining of myosin on hMSCs cultured under the same conditions of panel a, co‐stained with ActinGreen and DAPI. c) Scatter plots showing the ratio of myosin/actin on hMSCs cultured under the conditions of panel b (mean ± SD, *n* = 50). d) Time‐lapse images of hMSC‐GFP upon the treatment of **P3** (200 µm). e) Scatter plots representing the ratio of hMSC length/width with or without the treatment of **P3** (200 µm) (mean ± SD, *n* = 50). f) Scattering plots representing the ratio of YAP intensities in the nucleus relative to that in the cytoplasm of hMSCs with or without the treatment of **P3** (200 µM) for different periods of time (mean ± SD, *n* = 50). *p < 0.1, **p < 0.01, ***p < 0.001, ****p < 0.0001.

To understand the correlation between extracellular scaffold formation and the phenotypic response of hMSCs following the treatment with peptide **P3**, we analyzed the transformation of focal adhesions (FAs) and the reorganization of the cytoskeleton in hMSCs. Immunofluorescence (IF) staining of paxillin on the hMSCs treated with **P1** or **P2** or cultured on fibronectin coated surface, revealed small punctate FAs colocalized with the ends of stress fibers (**Figure** **3**A). Conversely, upon treatment with **P3**, FAs were large and elongated, distributed throughout the ventral cell surface, not just colocalized with the ends of stress fibers but also connected to many small F‐actin bundles spanning the cell indicating. Such enriched FAs and larger FAs both indicate stable and strong adhesion strength (Figure 3A).^[^ ^23^ ^]^ To correlate these different adhesion strengths with mechanotransduction‐mediated osteogenesis, myosin localization patterns were visualized through IF staining. As depicted in Figure 3B, only upon treatment with **P3** did myosin assemble into thick filaments colocalized with F‐actin. Together with the highest myosin/actin ratio (Figure 3C) and the graduate cell elongation (Figure 3d,e; Figure S21a, Supporting Information), demonstrated that the self‐assembly of **P3** on the apical membrane elongates hMSCs and induces higher contraction force than both fibronectin coating and mono‐targeting assembling peptides. Concurrent with this mechanotransduction,^[^ ^24^ ^]^ YAP translocate to the nucleus (Figure 3f; Figure S21b,c, Supporting Information), leading to a much stronger promotion effect on osteogenesis.^[^ ^25^ ^]^

**Figure 4:**
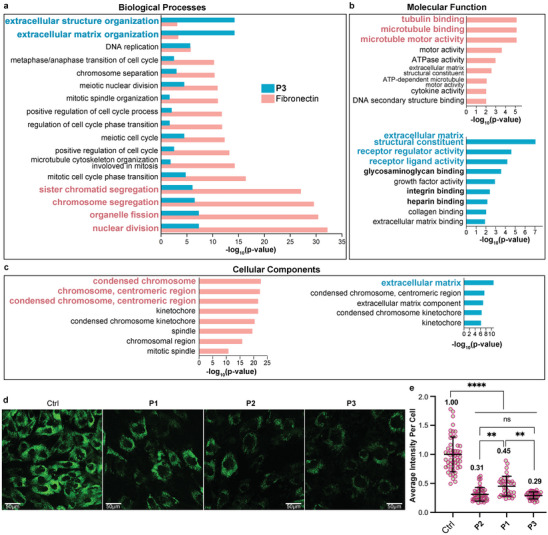
**P3** treatment induces ECM organization and receptor regulation via integrin/HSPG binding. Significant GO terms of associated biological processes a), molecular function b), and cellular components c) from differentially regulated genes of hMSCs cultured on fibronectin coated surface or treated by **P3** (*p* < 0.05). Immunofluorescence (IF) staining of Chordin on hMSCs d) and scatter plots of average intensity e) upon the treatment of **P1**, **P2**, and **P3** at a concentration of 200 µM for 21 days (mean ± SD, *n* = 50). *p < 0.1, ** *p* < 0.01, ***p < 0.001, ****p < 0.0001.

To comprehensively decipher the cellular response to **P3**‐induced phenotypic elongation and substantiate the mechanotransduction‐mediated promotion of osteogenesis, we employed whole‐transcriptome sequencing (RNA‐seq) to unveil genome‐wide changes in gene expression. The RNA‐seq analysis of hMSCs, conducted after a 14‐day cell culture under **P3** treatment and fibronectin assay, provided a panoramic insight into diverse cellular processes, encompassing responses to external stimuli, proliferation, and differentiation.^[^ ^26^ ^]^ In the fibronectin assay, key Gene Ontology (GO) terms significantly enriched in Biological Processes (BP) included nuclear division, organelle fission, chromosome segregation, and sister chromatid segregation (**Figure** **4**a). Molecular Functions (MF) exhibited enrichments in tubulin binding, microtubule binding, and microtubule motor activity (Figure 4b), while Cellular Components (CC) featured condensed chromosome, chromosome, and centromeric region (Figure 4c), all indictive of cell cycle‐related activities. In contrast, **P3** treatment resulted in significant GO enrichments related to ECM and membrane proteins. Specifically, key BP terms included extracellular structure organization and extracellular matrix organization; CC featured extracellular matrix; and MF showed enrichments in extracellular matrix structural constituent, receptor regulator activity, and receptor ligand activity. Notably, highly enriched MF terms comprised glycosaminoglycan binding, integrin binding, and heparin binding (Figure 4b), aligning with the integrin/HSPG dual targeting design of peptide **P3**. Overall, the GO enrichment analysis suggests that the **P3**‐assembled extracellular scaffold regulates ECM organization through binding with integrin and HSPG on hMSCs, indicating reciprocal interactions between the extracellular scaffold and hMSCs. This elucidates how hMSCs mediate the construction of extracellular scaffolds via the instructive assembly of **P3**.

While the enhancement of osteogenesis through integrin‐binding‐induced mechanotransduction has been extensively studied, the impact of HSPG‐binding on osteogenic differentiation remains less explored. Given the adhesion of **P3** assemblies to the apical membrane of hMSCs, we conducted immunostaining for Chordin on hMSCs. Chordin binds to cell‐surface HSPG, such as syndecans, rather than to basement membrane HSPG containing the perlecans. Interactions between Chordin and HSPGs have been shown to strongly antagonize BMP signaling.^[^ ^27^ ^]^ Notably, **P3**‐treated hMSCs exhibited the lowest average fluorescence intensity (Figure 4d,e), indicating the lowest Chordin‐HSPG binding rate among all treatments. This includes integrin‐binding peptide **P1** and HSPG‐binding peptide **P2**, both of which form extracellular scaffolds on the apical membrane of hMSCs. Collectively, these experimental results suggest that integrin/HSPG‐binding peptide **P3** assemblies effectively suppress Chordin‐HSPG interactions, thereby potentiating BMP signaling resulting into a promotion of osteogenic differentiation.

**Figure 5:**
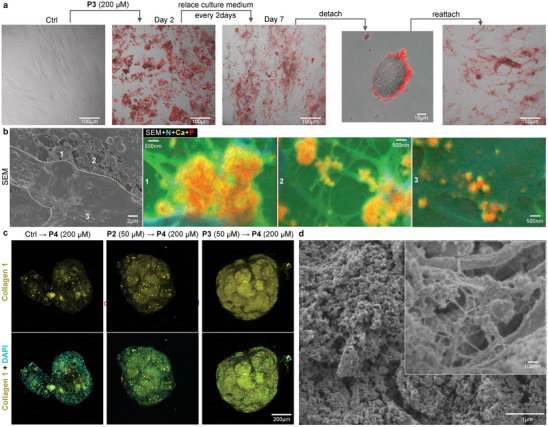
Adaptivity of extracellular scaffold and in situ biomineralization via sequential PBIPA. a) Overlay of bright field and fluorescent images of hMSCs stained with Congo Red upon sequential treatment of **P3** and **P4**, followed by cell detachment and reattachment. b) SEM image and the correlated zoom‐in EDS layered images of hMSC surface sequentially treated by **P3** (200 µM) and **P4** (200 µM) during osteogenesis. c) The Z‐stacks of IF staining of Collagen I (yellow), Fibronectin and Laminin on hMSC spheroids upon the treatment of **P1**/**P4**, **P2**/**P4**, and **P3**/**P4** for 21‐day osteogenesis and co‐stained with DAPI (cyan). d) SEM image of hMSC spheroid upon the treatment of **P3**/**P4** for 21‐day osteogenesis.

To validate the adaptive characteristics of the integrin/HSPG‐instructed peptide‐assembled extracellular scaffold, the culture medium of **P3**‐treated hMSCs underwent replacement every 2 days without replenishing **P3**. Remarkably, the **P3** assemblies rely on physical interactions persisted in their adhesion to hMSCs, demonstrating such robust adhesiveness that they attached to detached hMSCs after trypsinization. Following reattachment, the **P3** assemblies were successfully maintained on hMSCs (**Figure** **5**a). This adhesive extracellular scaffold represents a distinctive form of adaptability, reforming itself following changes in cell shape. To comprehensively evaluate the stepwise formulation during osteogenic differentiation, **P4** was introduced to hMSCs pre‐cultured with **P3**, major building blocks of **P3** including **P5** and a mixture of **P6**/**P7**. Importantly, no cytotoxicity was induced (Figures S22–S24, Supporting Information), affirming the excellent biocompatibility, a crucial attribute for applications in tissue engineering. The sequential administration of **P3** and **P4** during osteogenic differentiation triggered in situ deposition and accelerated growth of CaP infiltrating into the extracellular fibrous networks (Figures S25 and S26, Supporting Information), as an adaptive response to osteogenic differentiation. Additionally, small CaP particles were observed accumulating on the apical membrane (Figure 5b; Figure S27, Supporting Information) indicating the formation of a bony microenvironment. Taking advantage of the adaptive characteristics of hMSC‐mediated construction via sequential PBIPA, the **P3** and **P4** were introduced sequentially to 2D cell culture to generate biphasic scaffolds first, following a trypsinization and u‐dish 3D culturing to form hMSC spheroid with interpenetrated biphasic scaffold. In line with qPCR analysis (Figure 2c), an upregulation in collagen expression was evident in z‐projection of IF imaging. Eventually, the constructed scaffold enriched with CaP and collagen, aligns with the complexities of native bone tissue, offering tightly packed hMSCs spheroids (Figure 5c) with interpenetrated biphasic scaffold (Figure 5d).

**Figure 6:**
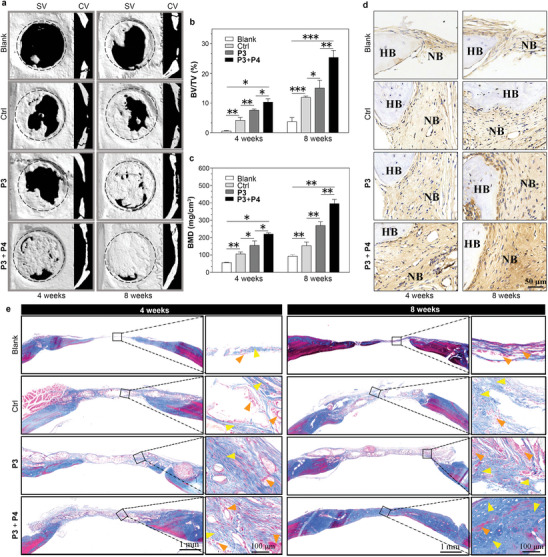
Adaptive biphasic scaffolds promote bone regeneration. a) Representative micro‐CT images of calvarial defects upon different treatments: blank, control (Ctrl), **p3** and **P3** + **P4**. The micro‐CT images including a superfical view (SV) and a coronal view (CV), were taken at 4‐ and 8‐weeks post‐implantation. Quantitative analysis of bone parameters from the micro‐CT images including bone tissue volume to total tissue volume (BV/TV) b) and bone mineral density (BMD) c). d) Immunohistochemistry staining of osteocalcin (OCN) in calvarial defects after implantation of blank, ctrl, **P3** and **P3** + **P4** for 4 and 8 weeks (HB: host bone; NB: new bone). e) Masson staining of calvarial decalcified sections of blank, ctrl, **P3** and **P3** + **P4** groups at 4‐ and 8‐weeks post‐implantation. (The mature bone was indicated by orange arrow, and new bone tissue was indicated by yellow arrows). Data shown as the mean ± SD (*n* = 3).

To access the efficacy of the adaptive scaffolds in promoting osteogenesis, we conducted in vivo bone regeneration experiments. Bone defects were created in the calvarium of rats (Figure S28, Supporting Information), and spheroids of treated hMSCs were implanted into the defect sites. After a specified period, bone samples were retrieved for radiological and histological analyses. X‐ray micro‐computed tomography (micro‐CT) images in **Figure** **6**a depict the defect sites after 4 and 8 weeks of implantation. In the blank group, minimal new bone formation was observed even after 8 weeks, whereas the control group, implanted with scaffold‐free hMSCs spheroids, and the **P3** group, implanted with **P3**‐treated hMSCs spheroids, exhibited regenerated bone tissues from the edge of the defects after 4 weeks. Notably, the **P3** group continued to show significant progress with substantial bone tissue filling the central cavity after 8 weeks. The most notable bone regeneration occurred in the **P3**+**P4** group, implanted with biphasic scaffold‐interpenetrated hMSCs spheroids. Four weeks post‐implantation, the defect sites displayed extensive regenerated bone tissue, and 8 weeks later, the defects were nearly fully healed.

To quantitatively assess bone regeneration, we evaluated several geometrical parameters, including bone volume faction (BTV) (Figure 6b), bone mineral density (BMD) (Figure 6c), trabecular thickness (Tb. Th), and trabecular number (Tb. N) (Figure S29, Supporting Information). Comparing the control, **P3** and **P3**+**P4** groups, the average BTV increased from 4.2%, 7.6% to 10.4% and from 12.0%, 15.1% to 25.4% for 4‐ and 8‐week healing periods. Simultaneously, the average BMD rose from 106.8, 155.9 to 221.3 mg cm^−3^ at 4 weeks and from 154.6, 270.6 to 396.5 mg cm^−3^ at 8 weeks. Furthermore, the **P3**+**P4** group exhibited the highest values for both Tb. Th and Tb. N. The morphometric analyses demonstrate that the **P3**+**P4** group excels in restoring bone volume, mineral density, and trabecular connectivity. Immunohistological staining of osteocalcin (OCN) revealed enhanced bone generation in the defect regions, with noticeably more brown staining in the **P3**+**P4** group than in the other groups (Figure 6d). Masson staining (Figure 6e) and H&E staining (Figure S30, Supporting Information) images further supported these findings, showing increased newly formed bone and tissue regeneration in the **P3**+**P4** group than in the other groups. Four weeks after implantation, bulk bone structures covered the middle of the defect cavity, and after 8 weeks, the new bone nearly enveloped the entire cranial defect. Additionally, abundant connective fibrous tissue along with dispersive osteoid masses and osteocytes were observed in **P3**+**P4** group. These results demonstrate the greatly improved bone regeneration potential of the adaptive scaffold when in synergy with osteogenic differentiation.

## 3 Conclusion

In summary, our development of adaptive biphasic scaffolds through sequential protein‐instructed peptide assembly represents a pioneering stride in the pursuit of biomimetic materials for materials‐based cell therapy. By leveraging stage‐specific proteins and employing in situ molecular assembly, our approach ensures a customized dynamic response to the everchanging cellular microenvironment, crucial for steering stem cell fate throughout differentiation, continuously mirroring the intricate cues inherent in natural tissue development. Through adherence to the stem cell membrane, the scaffolds demonstrate remarkable adaptability to everchanging cell types across osteogenic differentiation, positioning them as a superior candidate surpassing established methods and strategies for bone healing. While our current study primarily focuses on osteogenic differentiation, the underlying principles of sequential protein‐instructed peptide assembly hold potential for extrapolation to other tissue types and regenerative applications. The modular nature of our approach allows for customization, rendering it adaptable to diverse tissue engineering scenarios and cell‐based therapies. This not only holds promising implications for enhancing therapeutic outcomes but also paves the way for personalized medicine approaches tailored to individual patient needs. Future exploration may unveil its potential in treating a spectrum of tissue defects and degenerative conditions, thereby revolutionizing the landscape of regenerative therapies.

In conclusion, this research not only advances the fields of biomaterials and tissue engineering but also offers promising solutions for improving patient outcomes and addressing unmet clinical needs in regenerative medicine. The integration of molecular self‐assembly, molecular co‐assembly, and biomarker protein‐instructed molecular assembly into our methodology represents a paradigm shift in scaffold design, emphasizing nanoarchitectonics principles, with profound implications for the future of regenerative medicine.

## 4 Experimental Section

### Materials for Peptide Synthesis

The Fmoc‐amino acids and resin utilized in the peptide synthesis were purchased from GL Biochem (Shanghai) Ltd. China. Reagents including DIPEA, HBTU, NMP, NMM, and TFA, as well as solvents such as DCM, MeOH, acetone, diethyl ether, and acetonitrile, were purchased from Sigma, Nakalai and WAKO (Japan).

### TEM Imaging of Peptides in Solution

Carbon‐coated copper grids were employed for TEM sample preparation. These grids were discharged to enhance their hydrophilicity. Subsequently, 5 µL of the sample solution was dropped onto the grid. After 10 s, excess solution was removed using filter paper. The grids were then washed three times with 5 µL of distilled water, followed by removal of excess water. The samples were stained with 5 µL of 1% uranyl acetate for 10 s. After removal of excess uranyl acetate solution and air‐drying for another 10 s, the morphology of the molecular assembly was observed under high vacuum using a TEM (JEM‐1230R, JEOL, Japan).

### SEM Imaging of Peptides in Solution

The sample solutions were dispensed onto a 35 mm glass‐bottom dish. Subsequently, they were frozen at ‐80 °C for 4 hours. Then the samples underwent overnight freeze‐drying using a freeze‐dryer (ES‐2020, Hitachi, Japan). For SEM observation, the freeze‐dried samples were coated with platinum using a sputter coater (E‐1030, Hitachi, Japan) and examined with a SEM (JSM‐7900F, JEOL, Japan) at an operating voltage of 3 kV.

### SEM Imaging of Peptide‐Treated hMSC Cells

Following peptide treatment, the culture medium was aspirated, and the cells were washed three times with 1xPBS buffer. Subsequently, the cells were fixed with 2.5% glutaraldehyde in 0.1 m cacodylate buffer for 30 mins, followed by further fixation with 1% OsO_4_ in 0.1 m cacodylate buffer for 30 min. The cells were then washed using Milli‐Q water for 5 min repeated 3 times, progressively dehydrated using a graded series of ethanol, rinsed with t‐BuOH, and freeze‐dried in a lyophilizer (Freeze Dryer, Labconco) for over 12 h. Prior to imaging, all samples were coated with a 5 nm osmium layer using osmium coater OPC80T (Filgen). SEM images were aquited using an ultra‐high‐resolution FE‐SEM JSM‐IT800SHL (JEOL, Japan) at an operating voltage of 1.0 kV.

### EDS Mapping and Imaging

Scanning Electron Microscope Energy Dispersive Spectroscopy (SEM‐EDS) measurements were conducted using Oxford Instruments EDS system (Ultimax 170 mm^2^ x2). All samples were examined at 5.0 kV with the working distance of 10 mm and a frame Number of 20 – 30 scan for 3 to 4 min. Elemental mapping was performed to determine the spatial distribution of nitrogen (N), carbon (C), oxygen (O), phosphorus (P), and calcium (Ca).

### CD Spectroscopy

CD spectra were recorded at room temperature using a JASCO J‐820 spectrometer. The bandwidth was set at 1.0 nm and the measurement range extended from 190 to 250 nm. All measurements were carried out in a quartz cuvette with a 1 mm path length. Theoretical curves were generated by summing the comtributions from each individual compound.

### Cell Culture of hMSCs

Human mesenchymal stem cells (hMSCs) at passage 2 were procured from Lona (Walkersville, MD; Material No. PT‐2501, Batch No. 19TL68853), and subcultured twice in 75 cm^2^ tissue culture treated flasks using Mesenchymal Stem Cell Basal Medium (MSCBM) (Lonza, Walkersville, MD) to expand the cell number. The cells at passage 4 were harvested by treatment with 0.25% trypsin/EDTA (Sigma, USA) and utilized for subsequent experiments. Osteogenic differentiation was induced using osteogenic medium, with medium refreshment every 2 days. The osteogenic medium comprised complete Dulbecco's Modified Eagle Medium (DMEM, low glucose) supplemented with 10% fetal bovine serum (FBS), 10 nm dexamethasone, and 10 mm β‐glycerolphosphate.

### Cell Spheroid Culture

Human mesenchymal stem cells (hMSCs) at passage 4 were grown in 75 cm^2^ tissue culture treated flasks in MSCBM (Lonza, Walkersville, MD) until reaching 80% confluency. Subsequently, the cells were treated with P3 (KRSRFFFIKLLI, 50 m) in the osteogenic medium for 24 h. The culture medium was replenished every 2 days. On the 7th day of osteogenic differentiation, P4 (NapFFYpE, 200 µm) was added to the osteogenic medium and co‐incubated with cells for 24 h, followed by two washes with prewarmed PBS buffer. On the 9th day, the cells were detached using 0.25% trypsin/EDTA (Sigma, USA) and seeded onto a 96‐well U‐shaped‐bottom microplate (Thermo Scientific #174925) in osteogenic medium. The medium was refreshed every 2 days until the hMSCs spheroids were harvested for subsequent experiments on specific days post‐spheroids formation. For in vivo studies, Bone Marrow Mesenchymal Stem Cells (BMSCs) (OriCell RASMX‐01001) obtained from Sprague Dawley (SD) rats were used instead of hMSCs following the same procedure. After a 9‐day culture in the 96‐well U‐shaped‐bottom microplate, the spheroids were collected for implantation.

### Cell Viability WST‐1 Assay

Cells were seeded onto a 96‐well flat‐bottom tissue culture plate (Corning, Falcon) at a density of 0.5 × 10^4^ cells cm^−2^ and incubated overnight to facilitate cell adhesion. The medium was then replaced with osteogenic medium containing various peptide compounds at concentrations ranging from 25 to 500 µM. As a control, cells were cultured in osteogenic medium supplemented with an equal volume of PBS. Subsequently, the cells were cultured for an additional 72 h, after which their viability was assessed using a WST‐1 assay kit (Roche Diagnostics, IN).

### Cell Viability Live/Dead Assay

For the live/dead assay, a portion of the aforementioned samples was subjected to further examination using calcein‐AM and propidium iodide (PI) staining reagents obtained from the Double Staining kit (Dojindo, Japan). Subsequently, fluorescence microscopy (Nikon, Eclipse Ts2R) was employed to capture images of the stained samples.

### Antibodies

The primary antibodies used for immunoflorecence staining were shown as follows: anti‐Paxillin (1:100, ab32084, Abcam), anti‐Myosin IIA (1: 100, ab138498, Abcam), anti‐YAP (1: 50, YAP (H‐9): sc‐271134, Santa Cruz), anti‐chordin (1:100, ab279579, Abcam). The secondary antibodies used include Goat Anti‐Mouse lgG H&L (Alexa Fluor® 488) (ab150113, Abcam, 1:1000), Goat Anti‐Rabbit lgG H&L (Alexa Fluor® 488) (ab150077, Abcam, 1:1000), Goat Anti‐Rabbit lgG H&L (Alexa Fluor® 568) (ab175471, Abcam, 1:1000), Donkey Anti‐Rabbit lgGH&L (Alexa Fluor® 647) (ab150075, Abcam, 1:1000), Goat Anti‐Mouse lgG H&L (Alexa Fluor® 568) (ab175473, Abcam, 1:1000), Goat Anti‐ Mouse lgG H&L (Alexa Fluor® 647) (ab150115, Abcam, 1:1000).

### Confocal Imaging

hMSCs were seeded in a 35 mm glass bottom culture dish at a density of 1 × 10^4^ cells per dish and incubated at 37 °C in 5% CO_2_ to allow cell attachment. Subsequently, the culture medium was refreshed with osteogenic medium suspended with 200 µ of peptide compounds. After 72 h of culture, the medium was removed, followed by being washed with pre‐warmed PBS. A freshly prepared solution of Congo Red at a concentration of 0.1 mg mL^−1^ in culture medium was added to cells, which were then incubated in a cell culture incubator for an additional 30 min. Following incubation, the cells were washed tree times with PBS and fixed with 4% paraformaldehyde (PFA) for 30 min. Nuclei and F‐actin were visualized by staining with 4′,6‐diamidino‐2‐phenylindole (DAPI) and phalloidin‐Alexa 488 (Invitrogen, Carlsbad, CA) for 30 min at room temperature.

Cells for immunofluorescence analysis were initially fixed in 4% paraformaldehyde for 30 min and then blocked with 3% BSA in PBST (PBS with 0.1% Triton X‐100) for 1 h. Subsequently, the cells were incubated with primary antibody diluted in PBS containing 1% BSA overnight at 4 °C. Afterward, the cells were washed three times with PBS to remove unbound antibodies. The secondary antibodies of mouse IgG FITC 561 (1:1000, Millipore), or rabbit IgG (H+L) Alexa Flour 647 (1:500, Abcam), were prepared in the same solution and applied to the cells 2 h at room temperature in the dark,. Following that, the cells were washed three times with PBS. Nuclei and F‐actin filaments were stained with DAPI and phalloidin‐Alexa 488 (Invitrogen, Carlsbad, CA) for 30 min at room temperature. Immunofluorescence images were captured using laser‐scanning microscopes (Nikon A1 and Zeiss LSM780).

### Time‐Lapse Imaging of Cell Shape

The hMSCs‐GFP cell line obtained from Cyagen (Lot NO.: 1511222131, Japan) was treated with used **P3** (200 µm) in MSCGM suspended with 10% FBS. Time‐lapse images were captured every hour for a duration of 12 h using IncuCyte Live Cell Analysis System. Subsequently, the time‐lapse images were analyzed and quantified using imageJ.

### YAP Imaging and Quantification

Immunofluorescence staining was performed using the primary antibody of YAP. The cells were co‐stained with DAPI and Phalloidin‐Alexa 488, and their fluorescence images were acquired using laser‐scanning microscopes (Nikon A1 and LSM780, Zeiss). The nuclear‐cytoplasmic distribution ratio (Nuc/Cyto ratio) of YAP was determined using ImageJ with the following equation:

(1) $$\mathrm{Nuc}/\mathrm{Cyto}\;\mathrm{Ratio}=\frac{I_{\mathrm{nuc}}/A_{\mathrm{nuc}}}{\left(I_{\mathrm{total}}-I_{\mathrm{nuc}}\right)/\left(A_{\mathrm{total}}-A_{\mathrm{nuc}}\right)}$$

I_nuc_ represents integrated intensity of YAP fluorescence in the nucleus; A_nuc_ represents the size of nuclear region; A_total_ represents the size of the entire cell; I_total_ represents the integrated intensity of YAP fluorescence in the entire cell.

### Alkaline Phosphatase (ALP) Staining and ALP Activity Measurements

hMSCs at passage 4 were seeded in tissue culture treated 24‐well plate at a density of 1 × 10^4^ cells per well in MSCBM. After 24 h of culture, the MSCBM in each well was replaced with osteogenic medium containing various compounds at a series of concentrations or PBS buffer as a control. The cells were cultured for 7 days with medium changes every 2 days. Following this, the cells were washed three times with pre‐warmed PBS buffer and used for ALP staining and ALP activity analysis. For ALP staining, the cells were fixed by immersion in 4% paraformaldehyde for 10 min, followed by three washes with PBS. Subcequently, the fixed samples were immersed in a working solution (0.1% naphthol AS‐MX phosphate (Sigma) and 0.1% fast blue RR salt (Sigma) in 56 mM 2‐amino‐2‐methyl‐1,3‐propanediol working solution (pH 9.9) (Sigma) at room temperature for 10 min to induce blue staining. After washing with PBS three times, the samples were observed under an optical microscopy. For ALP activity analysis, a SensoLyte pNPP alkaline phosphatase assay kit (Anaspec, USA) was utilized. Briefly, cells without fixation were washed three times with PBS, then scraped off the plates, and collected in a 1.5 mL centrifuge tube. Subsequently, 0.2% Triton X‐100 was added and incubated with the cell suspension at 4 °C for 10 min to lyse the cells. The lysates were centrifuged at 2500 × g for 10 min to collect the supernatant. The collected supernatant was further incubated with a p‐nitrophenyl phosphate (pNPP) substrate solution at 25 °C for 1 h, and the absorbance was measured using a microplate reader at 405 nm. A standard calibration curve was generated using an ALP standard solution to determine the ALP concentration. The relative ALP activity was normalized to the cell number in each well.

### Real‐Time PCR Analysis

The expression of osteogenesis‐related genes, including runt‐related transcription factor‐2 (*Runx2*), ALP, secreted phosphoprotein 1 (*Spp1*), osteocalcin (*Ocn*), osteopontin (*Opn*), and osterix (*Osx*) was analyzed by real‐time PCR. After 21 days of culture in an osteogenic medium, the total RNA was extracted from hMSCs using Sepasol solution (Nacalai Tesque, Japan) following the manufacturer instructions. Complementary DNA (cDNA) was synthesized from 1 µg of purified total RNA using the iScript cDNA Synthesis Kit (Bio‐Rad). The synthesized cDNA served as a template for subsequent real‐time PCR analysis. The PCR reaction was performed in a 20 µL solution containing 10 µL of power SYBR Green PCR Master Mix (Applied Bio‐systems, Irvine, CA, USA), 7.4 µL of nuclease‐free water, 1.6 µL of cDNA solution, and 1 µL of primer solution. A QuantStudio 3 Real‐time PCR System (Applied Biosystems, Irvine, CA, USA) was employed with 40 cycles of amplification. The primers used for real‐time PCR are listed in Table S1 (Supporting Information).

### RNA‐Seq Analysis

Cells were seeded in 6‐well plates and treated with **P3** or fibronectin for 14 days. Then, the cells were harvested, and total RNA was extracted using TRIzol reagent (ThermoFisher, USA). RNA‐seq was performed by the Novogene Co., Ltd. Differential expression analysis was performed using DESeq2 R package. Genes with an adjusted P value <0.05 as determined by DESeq2 were assigned as differentially expressed. The identified gene sets were categorized based on Gene Ontology (GO) annotation, covering three main categories: biological process, cellular component, and molecular function. GO enrichment analysis of the differentially expressed genes was carried out on the GOC website (https://geneontology.org/). GO terms with a corrected P value < 0.05 were considered to be significantly enriched by the differentially expressed genes.

### Ethics Approval Statement

All experimental procedures involving animals were conducted with the approval of the Animal Ethics Committee of Zhengzhou University (approval number: ZZU‐LAC20221111[20]). All animal procedures adhered strictly to the guidelines outlined in the “Guide for the Care and Use of Laboratory Animals” of the National Research Council (US) (2011). Essential measures were implemented to minimize the suffering of laboratory animals, and strict control was maintained over the total number of animals used in the study.

### Surgical Procedures in Animal Models

Female Sprague Dawley (SD) rats, aged 11–12 weeks and weighing 250 ± 20 g, were purchased from HFK Bio‐Technology Co., Ltd. (Beijing, China). Ethical approval for all experimental procedures was obtained from the Animal Care and Experiment Committee of Zhengzhou University. The SD rats were randomly allocated into four groups: a blank group (*n* = 12) receiving no treatment, a control (Ctrl) group (*n* = 12) undergoing implantation of BMSC spheroids, a **p3** group (*n* = 12) undergoing implantation of **p3**‐treated BMSC spheroids, and a **p3**+**p4** group (*n* = 12) undergoing implantation of **p3** and **p4** sequentially treated BMSC spheroids. Prior to surgery, the SD rats were anesthetized with an appropriate dose of pentobarbital. After a brief period, the scalp was shaved, and circular defects with an 8 mm diameter were created on the calvaria using a trephine bur. Subsequently, the prepared samples were implanted into the defects. In the blank group, defects were left untreated. Following the surgical procedure, the wound was meticulously sutured and disinfected with iodophor. After 4 and 8 weeks, the SD rats were euthanized, and their bone samples were fixed in 10% neutral formalin (Macklin, China) for subsequent analysis.

### Micro‐CT and Histological Analysis

The harvested bone samples underwent micro‐CT (Bruker, USA) scanning to assess bone defect healing. Scanning was conducted with the following settings: 49 kV, 200 µA, and an AI 0.5 mm filter. Subsequently, 3D models were reconstructed using SCANCO Medical AG Visualizer software. To evaluate the bone healing process, calculations for the volume of newly formed bone relative to the total tissue volume (% BV/TV) and bone mineral density (BMD) were performed using Data Viewer software (Bruker, USA).

For histological analysis, the fixed samples were decalcified in a decalcifying solution for 48 hours and then dehydrated through a series of ethanol dilutions. Subsequently, the samples were embedded in paraffin and sectioned into 7 µm thick vertical cross‐sections. Tissue structure and specific components were visualized using Hematoxylin and Eosin (H&E), Masson's Trichrome (MT), and Osteocalcin (OCN) staining on the micro‐sections. The stained microsections were observed under an optical microscope (Carl Zeiss, Germany) to examine histological characteristics and access the bone defect healing process.

### Statistical Analysis

No statistical methods were used to predetermine the sample size. The experiments were not randomized, and the investigators were not blinded to allocation during experiments and outcome assessment. All measurements were performed on 1–3 biological replicates from separate experiments. The exact sample size and exact statistical test performed for each experiment are indicated in the appropriate figure legends. Statistical analyses were performed using GraphPad Prism (GraphPad Software, https://www.graphpad.com/). All bar graphs show mean values with error bars (s.e.m. or s.d., as defined in legends). The reported P values were corrected for multiple comparisons, where appropriate. Precise P values are shown in the figures and, when appropriate, are rounded to the nearest single significant digit. P values less than 0.0001 maybe be provided as a range. P values less than 0.05 are considered to be significant.

## Conflict of Interest

Y.Z. is an inventor on a patent application related to the data presented in this manuscript.

## Author Contributions

Y.C. and Y.Z. conceived the study and designed the experiments. Y.C., Q.Z., S.Y., G.L., C.S., X.H., S.A., N.A., and Y.Z. performed the experiments. Y.C. and Q.Z. conducted circular dichroism spectroscopy, TEM imaging, and cell viability assays. Q.Z. conducted NMR and mass spectroscopies. Y.C. and S.Y. maintained the mice and conducted the in vivo experiments. Y.C. and C.S. conducted qRT‐PCR, western blotting, and cell proliferation assays. Y.C., Q.Z., and X.H. conducted confocal microscopy. G.L. and Y.Z. conducted the RNA‐seq analysis. S.A. and N.A. conducted SEM imaging and EDS mapping. Y.Z. assembled the figures and wrote the manuscript, with editorial input from all authors.

## Supporting information

Supporting Information

## Data Availability

The data that support the findings of this study are available in the supplementary material of this article.
