# Supplementary material for: Formulate Adaptive Biphasic Scaffold via Sequential Protein‐Instructed Peptide Co‐Assembly
Source: Adv Sci (Weinh). 2024 May 24;11(29):2401478. doi: 10.1002/advs.202401478 (PMC11304238; doi:10.1002/advs.202401478)
Supplement: Supplementary file 1 — Supporting Information [file ADVS-11-2401478-s001.pdf]

ADVANCED SCIENCE
Open Access

Supporting Information

for *Adv. Sci.*, DOI 10.1002/advs.202401478

Formulate Adaptive Biphasic Scaffold via Sequential Protein-Instructed Peptide Co-Assembly

*Yazhou Chen, Qizheng Zhang, Shenyu Yang, Guanying Li, Chaochen Shi, Xunwu Hu, Shunsuke Asahina, Natsuko Asano and Ye Zhang**

Supplementary Information
Formulate Adaptive Biphasic Scaffold via Sequential Protein-Instructed Peptide-Co-Assembly

Yazhou Chen, Qizheng Zhang, Shenyu Yang, Guanying Li, Chaochen Shi, Xunwu Hu, Shunsuke Asahina, Asano Natsuko, and Ye Zhang*

Correspondence to: zhangye@sslab.org.cn

This PDF file includes:
Supplementary Figure S1 to S30
Supplementary Table S1

PCR are listed in Table S1. Triplicate samples were used for the analysis (n = 3).

Table S1. Primers for real time PCR analysis.

mRNA	Description	Oligonucleotide
Gapdh	Glyceraldehyde-3-phosphate dehydrogenase	Forward 5'- GCATCGTGGAGGGACTTATGA-3'<br>Reverse 5'- GGGCCATCCACAGTCTTCTG-3'
Runx2	Runt-related transcription factor-2	Hs00231692_m1
Alp	Alkaline phosphatase	Forward 5'-GACCCTTGACCCCCACAAT-3'<br>Reverse 5'-GCTCGTACTGCATGTCCCCT-3
Spp1	Secreted phosphoprotein 1	Forward 5'-CTCAGGCCAGTTGCAGCC-3'<br>Reverse 5'-CAAAAGCAAATCACTGCAATTCTC -3'
Ocn	Osteocalcin	Forward 5'-GAAGCCCAGCGGTGCA -3'<br>Reverse 5'-CACTACCTCGCTGCCCTCC -3

Peptides

Analyst	:YL		
Lot No.	:P190510-YS725634		
Column	:Diamonsil C18, 4.6*250mm, 5um		
Solvent A	:0.1%Trifluoroacetic in 100% Acetonitrile		
Solvent B	:0.1%Trifluoroacetic in 100% Water		
Gradient	:	A	B
	0.0min	27%	73%
	25.0min	52%	48%
	25.1min	100%	0%
	30.0min	Stop	
Flow rate	:1.0ml/min		
Wavelength	:220nm		
Volume	:20ul		

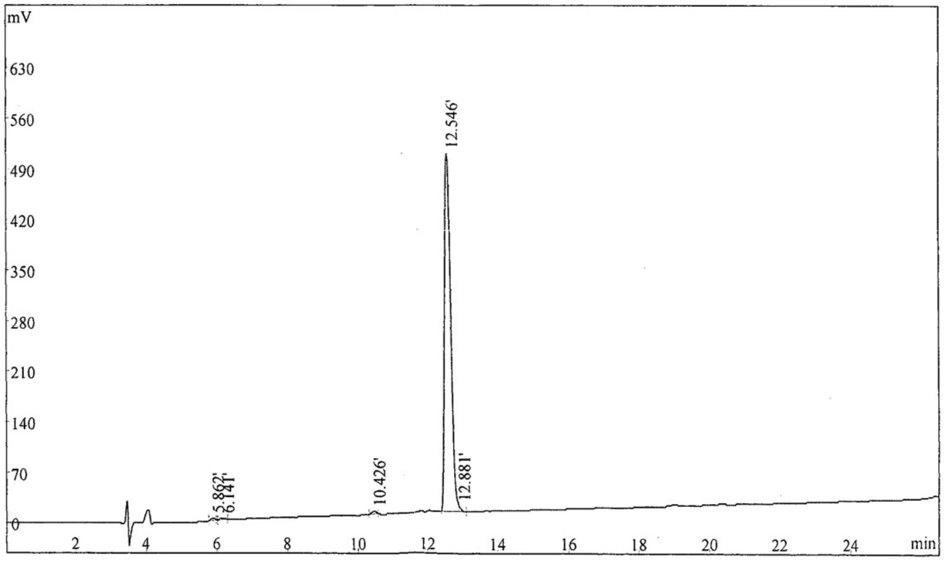

Rank	Time	Quantity	Area	Height
1	5.862	0.3826	21085	2938
2	6.141	0.3708	20436	2458
3	10.426	0.5356	29522	3611
4	12.546	98.24	5414921	494321
5	12.881	0.4678	25782	7777
Total		100	5511746	511105

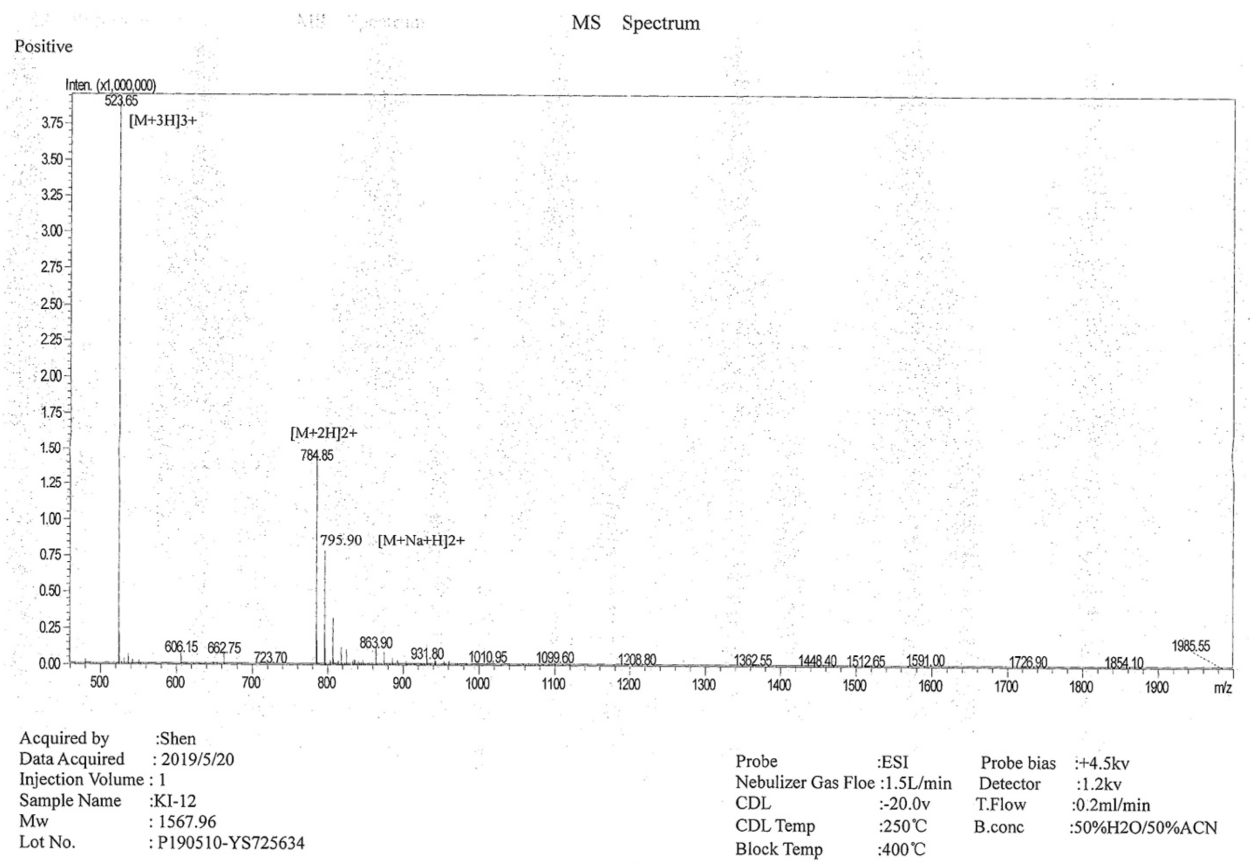

Figure S1. LC-MS spectra of peptide **P3** (KRSRFFFIKLLI).

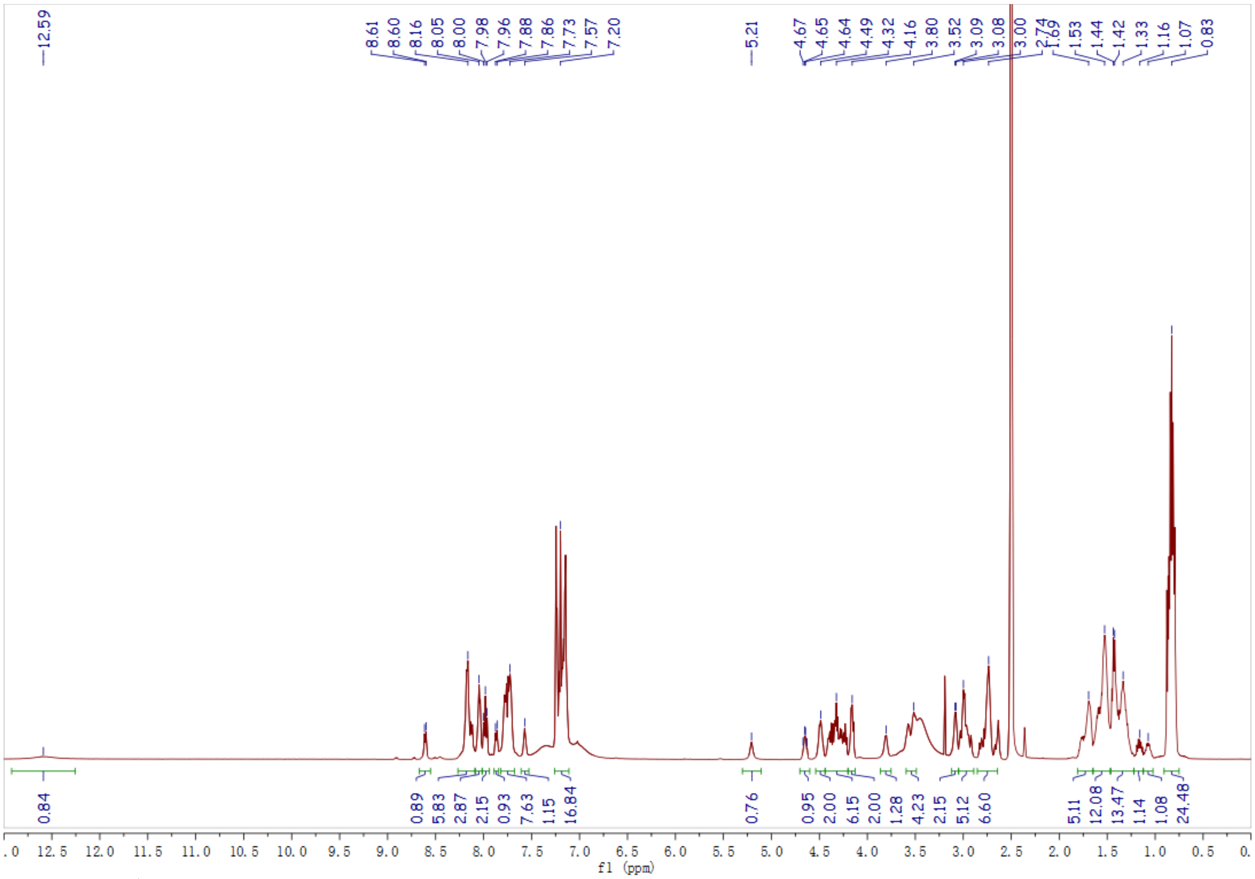

Figure S2. ^{1}H NMR spectrum of peptide **P3**.

^{1}H NMR (500 MHz, DMSO-d_6) δ 12.59 (s, 1H), 8.61 (d, J = 7.5 Hz, 1H), 8.26 – 8.09 (m, 6H), 8.08 – 8.01 (m, 3H), 7.98 (t, J = 9.1 Hz, 2H), 7.87 (d, J = 8.0 Hz, 1H), 7.82 – 7.68 (m, 1H), 7.61 – 7.54 (m, 1H), 7.27 – 7.11 (m, 15H), 5.21 (s, 1H), 4.65 (dd, J = 12.7, 8.2 Hz, 1H), 4.54 – 4.44 (m, 2H), 4.44 – 4.20 (m, 6H), 4.20 – 4.13 (m, 2H), 3.86 – 3.76 (m, 1H), 3.61 – 3.49 (m, 2H), 3.13 – 3.05 (d, J = 5.5 Hz, 2H), 3.05 – 2.89 (m, 5H), 2.85 – 2.65 (m, 7H), 1.81 – 1.65 (m, 5H), 1.65 – 1.47 (m, 12H), 1.46 – 1.23 (m, 13H), 1.21 – 1.12 (m, 1H), 1.12 – 1.02 (m, 1H), 0.91 – 0.75 (m, 24H).

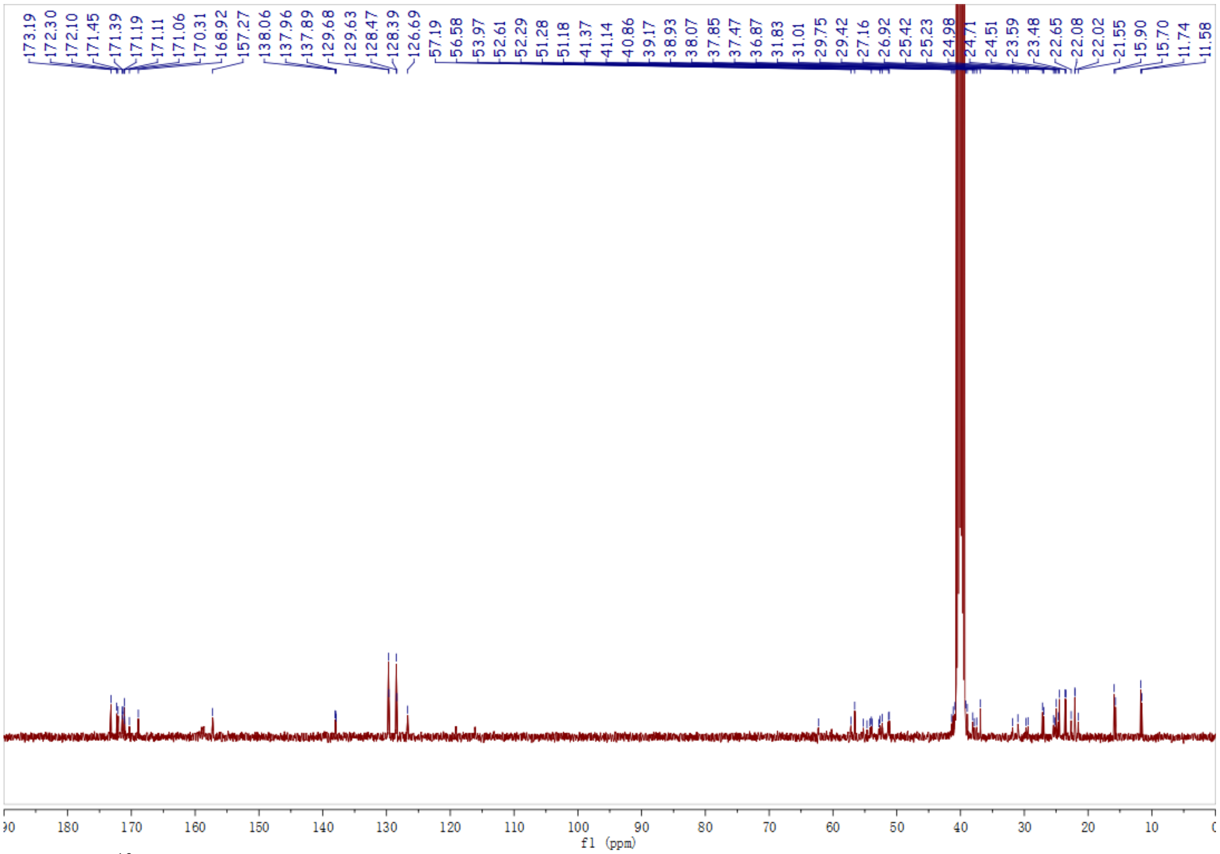

Figure S3. ^{13}C NMR spectrum of peptide **P3**.

^{13}C NMR (101 MHz, DMSO-d_6) δ 173.19, 172.30, 172.10, 171.45, 171.39, 171.19, 171.11, 171.06, 170.31, 168.92, 157.27, 138.06, 137.96, 137.89, 129.68, 129.63, 128.47, 128.39, 126.69, 62.27, 57.19, 56.58, 55.23, 54.65, 54.19, 53.97, 53.85, 52.82, 52.61, 52.29, 51.28, 51.18, 41.37, 41.14, 40.86, 39.17, 38.93, 38.07, 37.85, 37.47, 36.87, 31.83, 31.01, 29.75, 29.42, 27.16, 26.92, 25.42, 25.23, 24.98, 24.71, 24.51, 23.59, 23.48, 22.65, 22.08, 22.02, 21.55, 15.90, 15.70, 11.74, 11.58.

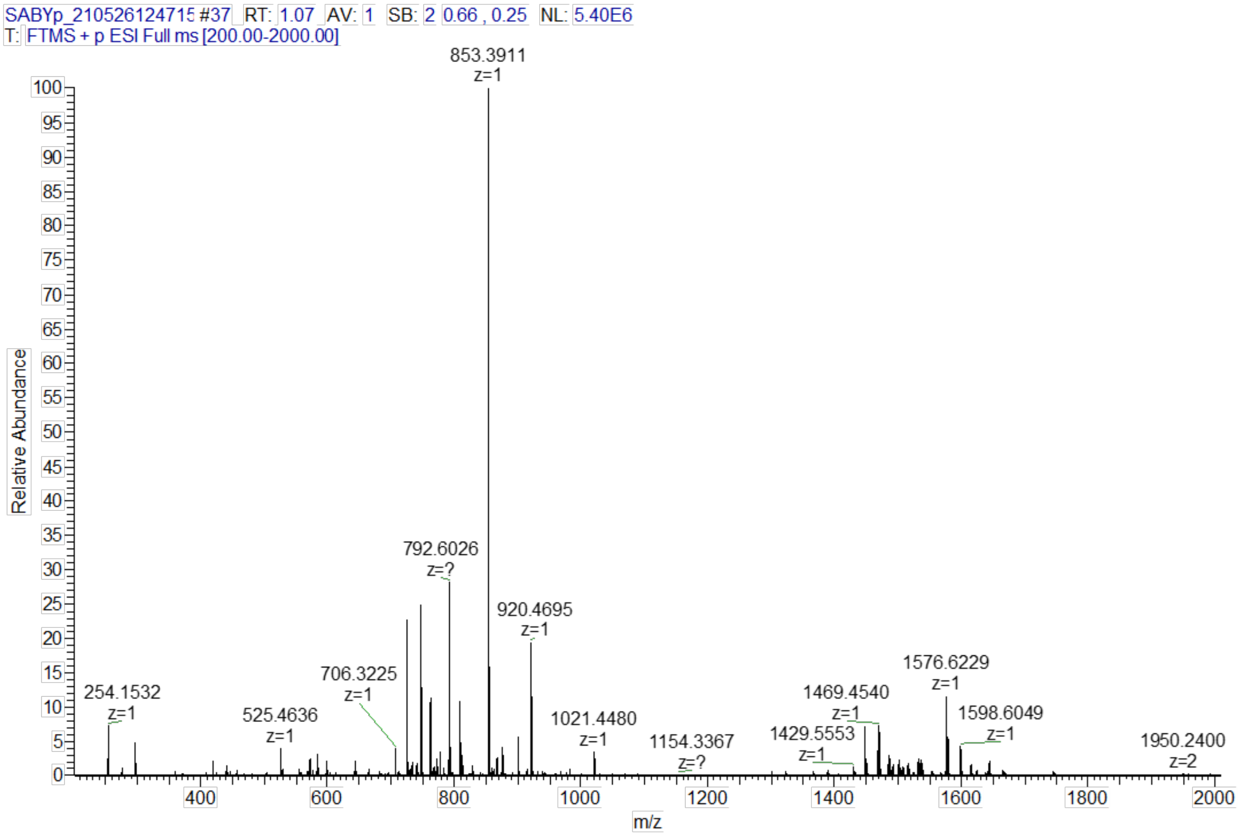

Figure S4. MS spectrum of peptide **P4** (Nap-FF$_{P}$YE).
ESI-MS (*m*/*z*) $[M+H]^+$ 853.3911.

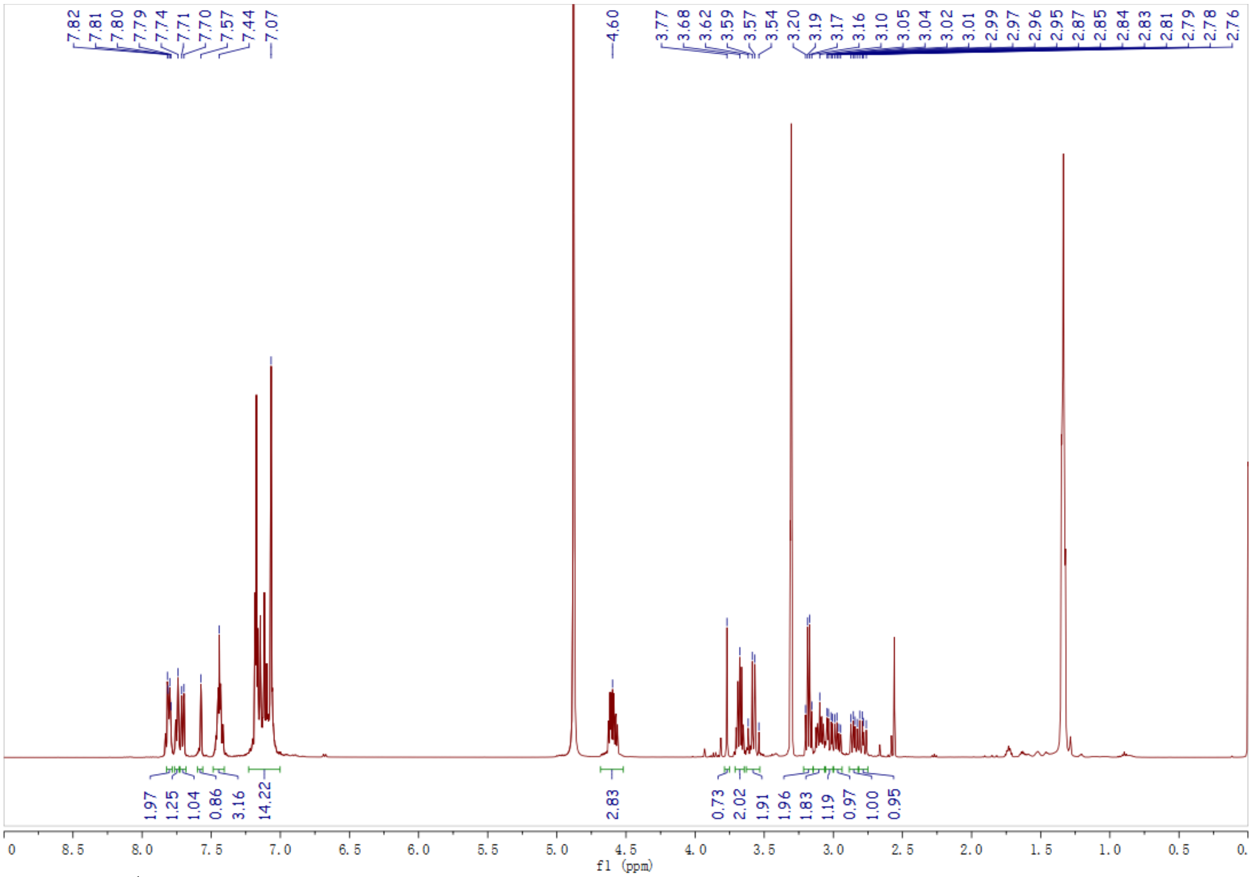

Figure S5. ^{1}H NMR spectrum of peptide **P4**.

^{1}H NMR (500 MHz, CD_3OD) *δ* 7.81 (dd, *J* = 9.4, 3.3 Hz, 2H), 7.76 – 7.73 (m, 1H), 7.71 (d, *J* = 8.4 Hz, 1H) , 7.60 – 7.56 (m, 1H), 7.48 – 7.41 (m, 3H), 7.23 – 7.01 (m, 14H), 4.68 – 4.52 (m, 3H), 3.77 (s, 1H), 3.71 – 3.64 (m, 2H), 3.58 (q, *J* = 14.4 Hz, 2H), 3.18 (q, *J* = 7.4 Hz, 2H), 3.14 – 3.05 (m, 2H), 3.03 (dd, *J* = 14.2, 4.8 Hz, 1H), 2.97 (dd, *J* = 14.0, 7.4 Hz, 1H), 2.85 (dd, *J* = 14.0, 8.8 Hz, 1H), 2.79 (dd, *J* = 14.0, 9.9 Hz, 1H).

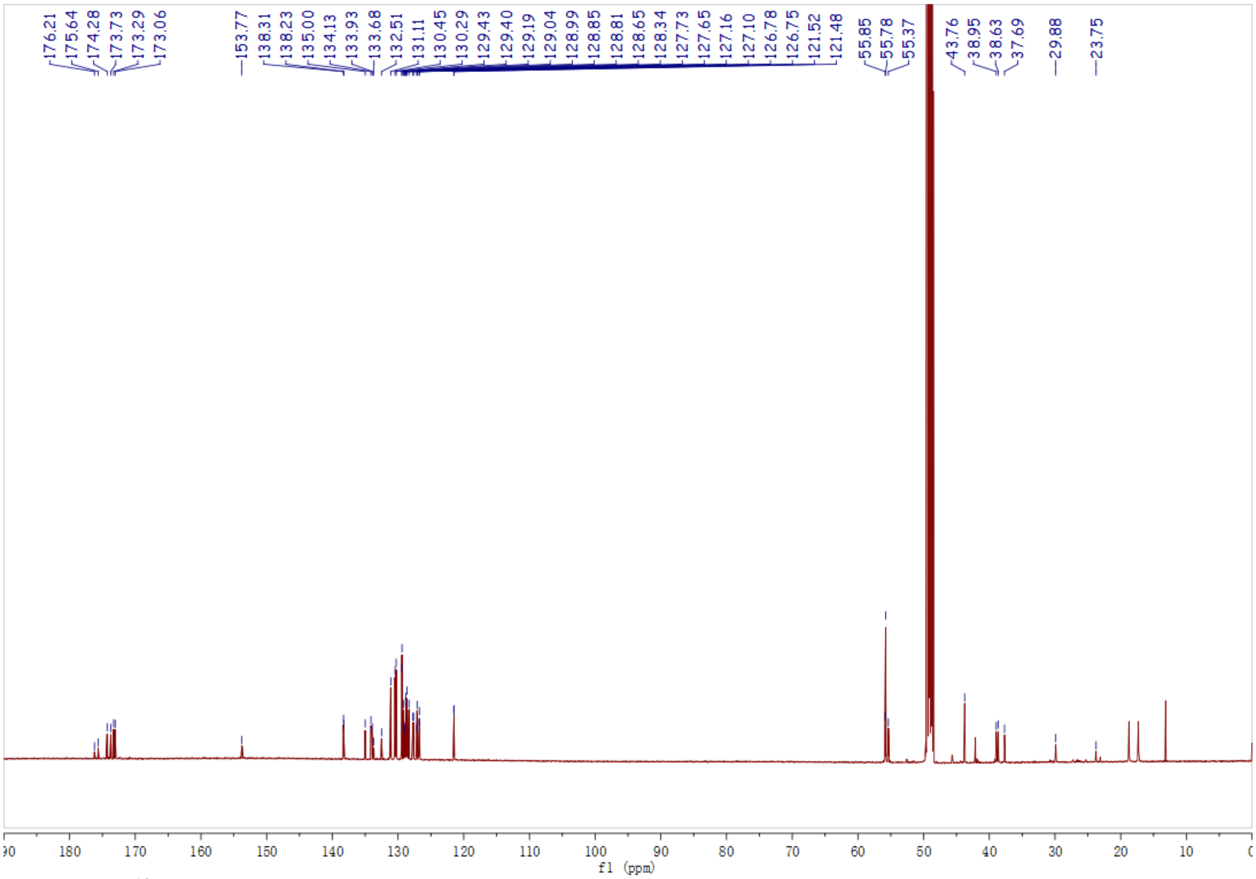

Figure S6. ^{13}C NMR spectrum of peptide **P4**.

^{13}C NMR (126 MHz, CD_3OD) δ 176.21, 175.64, 174.28, 173.73, 173.29, 173.06, 153.77, 138.31, 138.23, 135.00, 134.13, 133.93, 133.68, 132.51, 131.11, 130.45, 130.29, 129.43, 129.40, 129.19, 129.04, 128.99, 128.85, 128.81, 128.65, 128.34, 127.73, 127.65, 127.16, 127.10, 126.78, 126.75, 121.52, 121.48, 55.85, 55.78, 55.37, 43.76, 38.95, 38.63, 37.69, 29.88, 23.75.

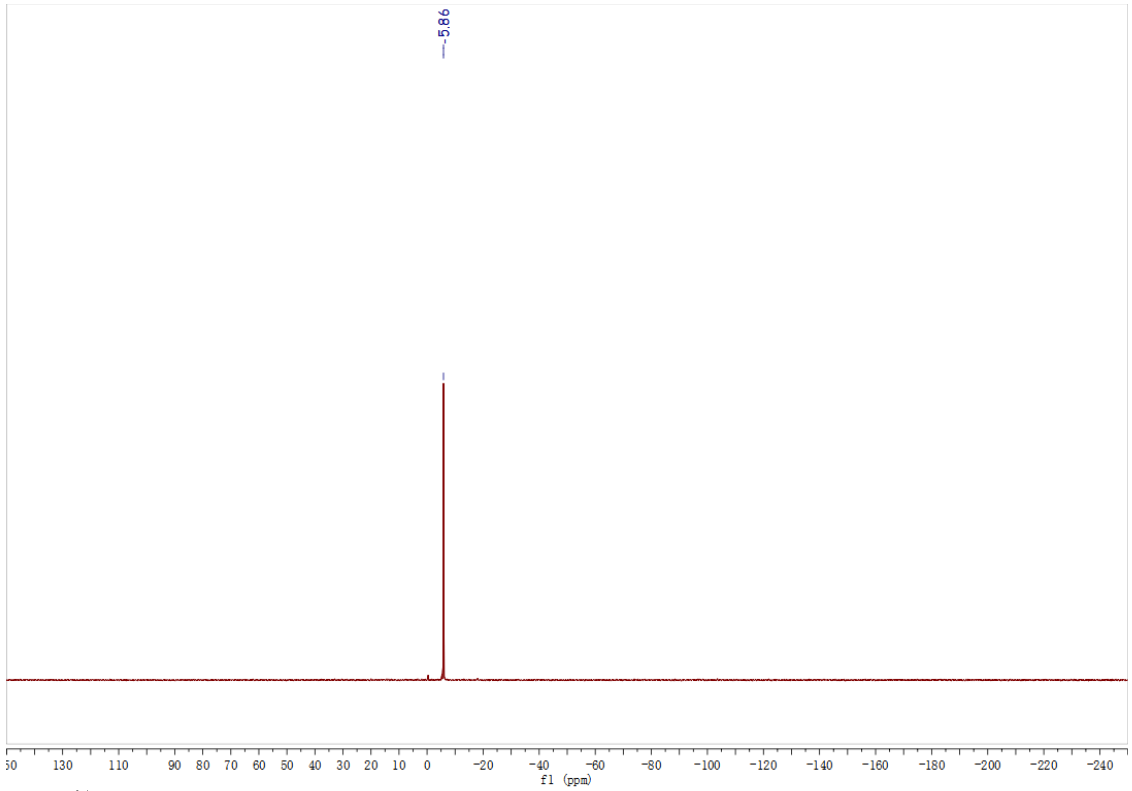

Figure S7. ^{31}P NMR spectrum of peptide **P4**.
^{31}P NMR (202 MHz, DMSO-d_6) δ –5.86.

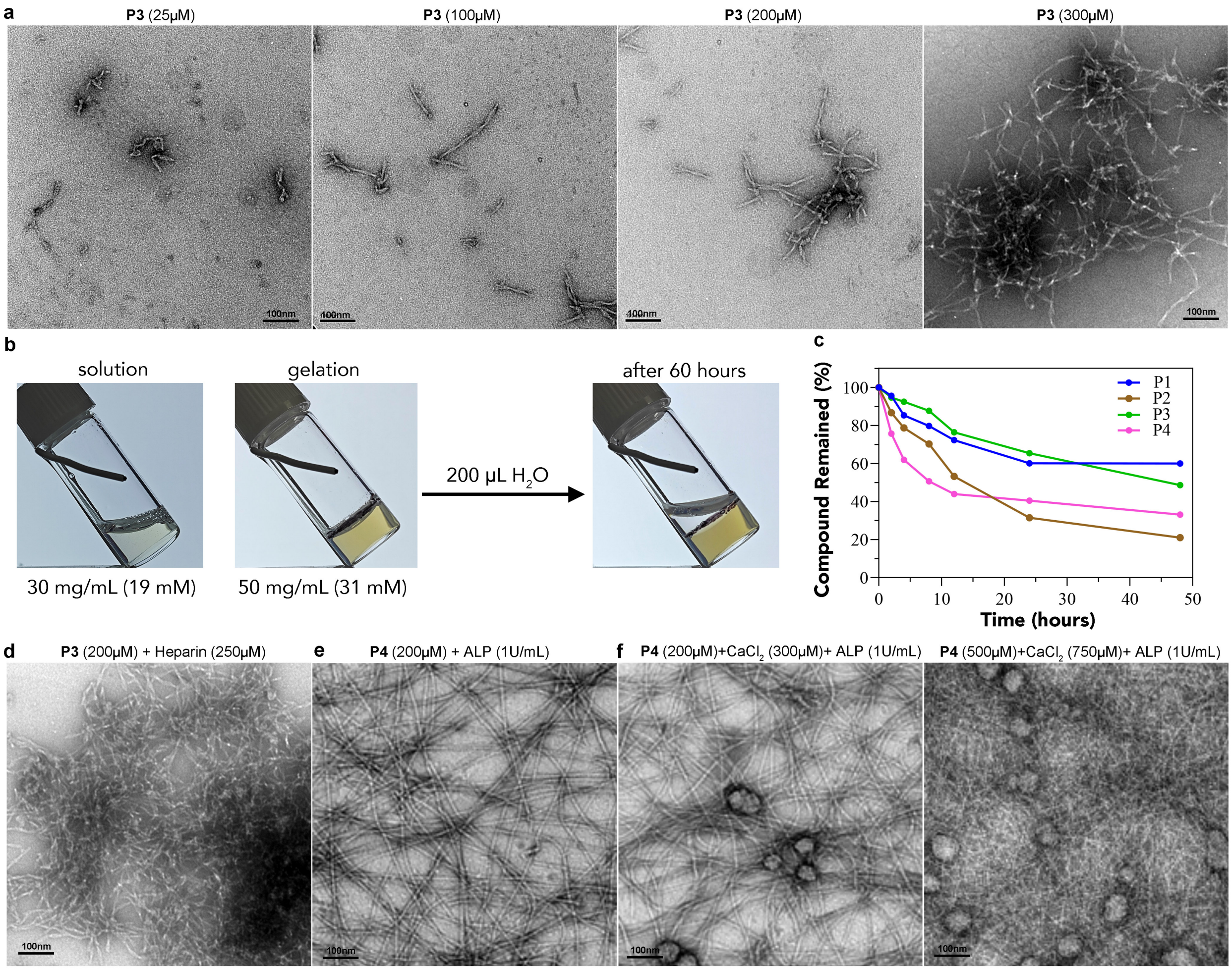

Figure S8. Assembly and gelation properties of peptide **P3** and **P4** under various conditions. **(a)** TEM images of **P3** in water at various concentrations. **(b)** Optical images of **P3** in water (pH = 7) at various concentrations. **(c)** Degradation curves of peptides **P1-4** (200μM) after treatment with proteinase H (3U/mL) for 48 hours. **(d)** TEM image of heparin and **P3** mixture, obtained by adding heparin (250 μM) to stabilized **P3** solution (200 μM). **(e)** TEM image of **P4** (200μM) after ALP (1U/mL) treatment in water. **(f)** TEM images of **P4** at 200μM and 500μM concentrations mixed with $CaCl_2$ at 300μM and 750 μM, respectively, after treatment of ALP (1U/mL) in water.

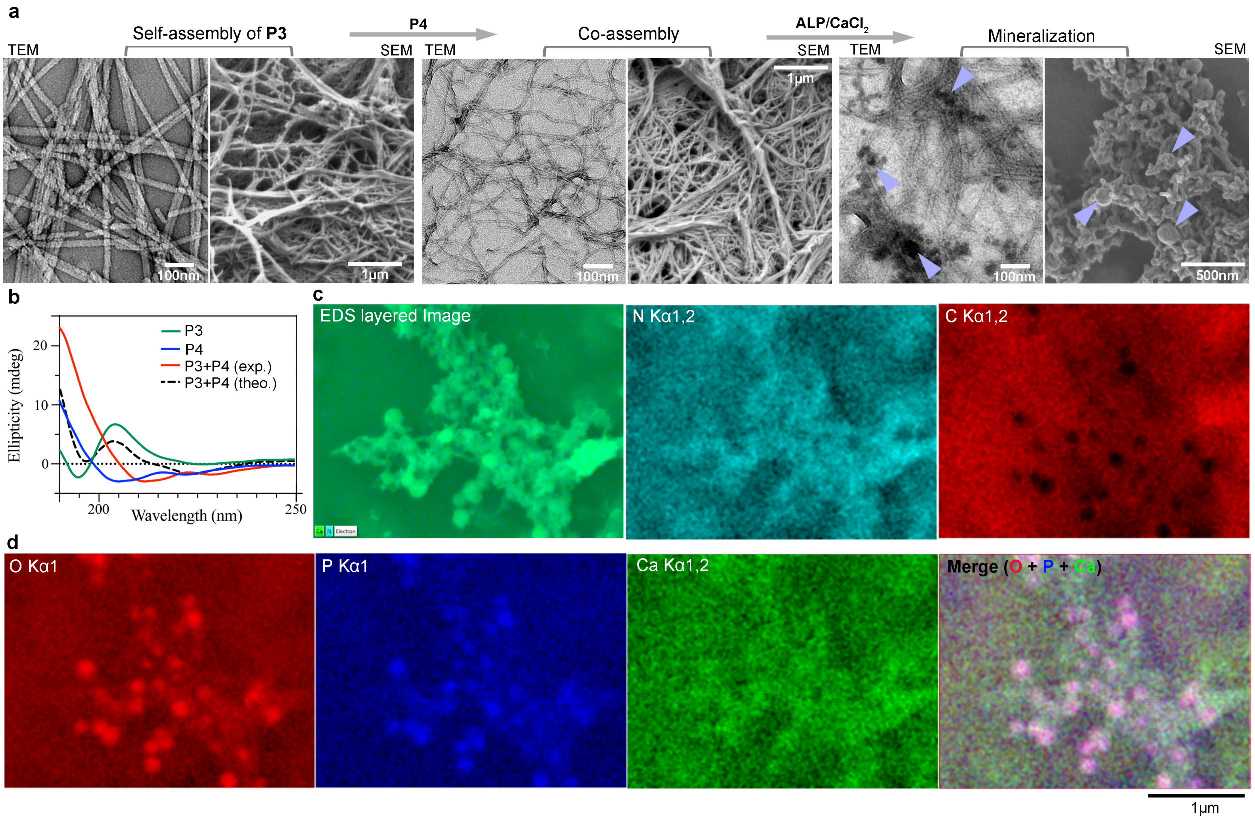

Figure S9. Construction of biphasic scaffold in aqueous solution. (**a**) TEM and SEM images of **P3** (200 μM) self-assembles in water, **P3** co-assembles with **P4**, and the following mineralization upon the treatment of ALP/$CaCl_2$. (**b**) CD spectra of **P3** (200 μM), **P4** (200 μM), mixture of **P3** and **P4** at 1:1 ratio in water. Exp. represents the experimental results, and Theo. represents the simple sum of single component CD spectra. (**c**) EDS layered image, and the correlated element mapping of N, and C of the nanostructures obtained at the final step in panel a. (**d**) Element mapping of O, P, Ca, and the overlay image of these three elements correlated to the EDS layered image in panel c.

P5 (Nap-FF)

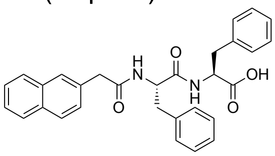

Figure S10. Chemical structure of peptide **P5**.

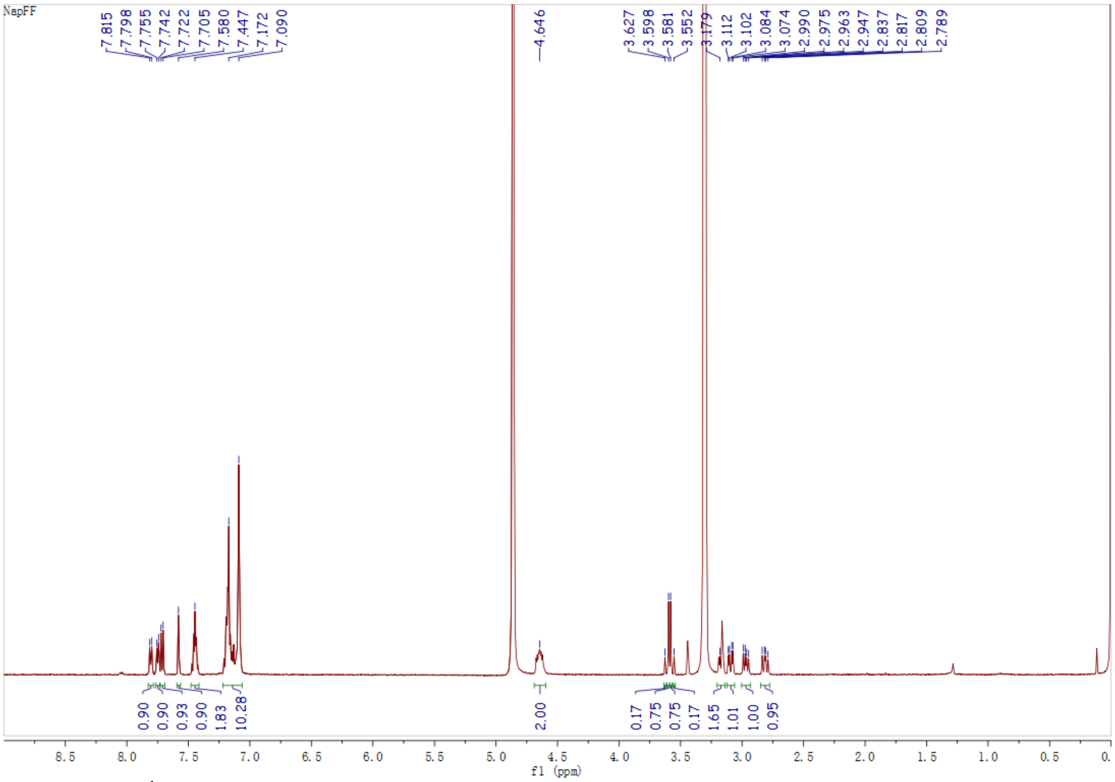

Figure S11. ^{1}H NMR spectrum of peptide **P5**.

^{1}H NMR (500 MHz, CD_3OD) δ 7.81 (d, J = 8.7 Hz, 1H), 7.75 (d, J = 6.9 Hz, 1H), 7.71 (d, J = 8.4 Hz, 1H), 7.58 (s, 1H), 7.48 – 7.41 (m, 2H), 7.22 – 7.06 (m, 10H), 4.69 – 4.60 (m, 2H), 3.59 (dd, J = 14.5, 8.5 Hz, 2H), 3.20 – 3.14 (m, 1H), 3.09 (dd, J = 14.0, 4.8 Hz, 1H), 2.97 (dd, J = 13.8, 8.1 Hz, 1H), 2.81 (dd, J = 13.9, 9.8 Hz, 1H).

P6 (IKLLI)

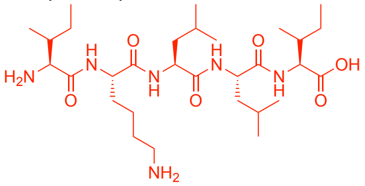

Figure S12. Chemical structure of peptide **P6**.

P7 (KRSR)

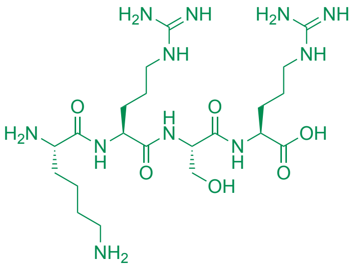

Figure S13. Chemical structure of peptide **P7**.

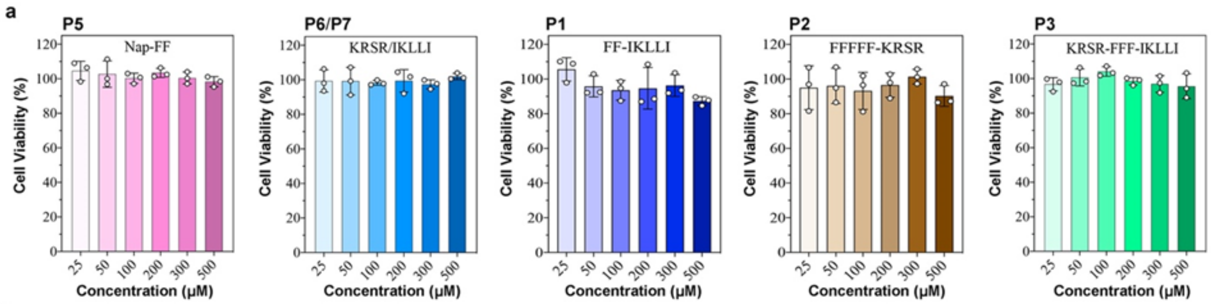

Figure S14. 3-day hMSCs viability upon the treatment of peptides **P1**, **P2**, **P3**, and control peptides **P5**, 1:1 mixture of **P6**/**P7** at various concentrations in basal medium (mean ± SD, n = 3).

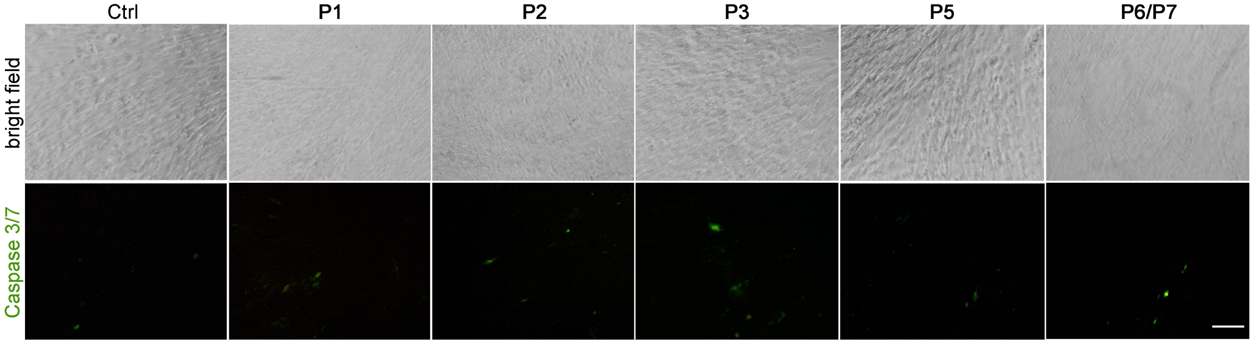

Figure S15. Bright field images and fluorescence images of hMSCs upon the treatment of peptides **P1**, **P2**, **P3**, and control peptides **P5**, 1:1 mixture of **P6**/**P7** at 200 μM in basal medium for 3 days and stained with caspase 3/7 (green). The scale bar represents 100 μm.

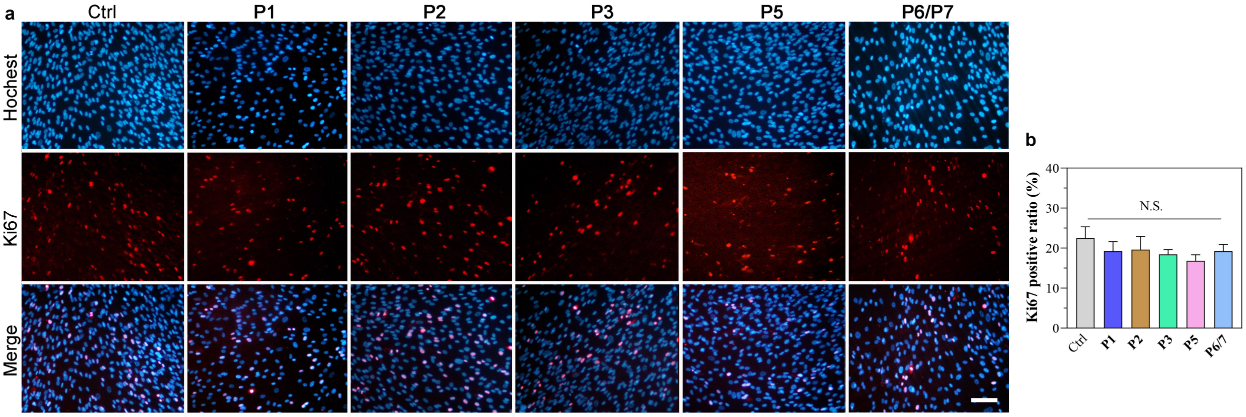

Figure S16. **(a)** Fluorescence images of hMSCs upon the treatment of peptides **P1**, **P2**, **P3**, and control peptides **P5**, 1:1 mixture of **P6**/**P7** at 200 μM in basal medium for 3 days and co-stained with hochest (blue) and Ki67 (red). The scale bar represents 100 μm. **(b)** Quantification of Ki67 positive ratio in hMSCs upon the treatments of panel a (mean ± SD, *n* = 3).

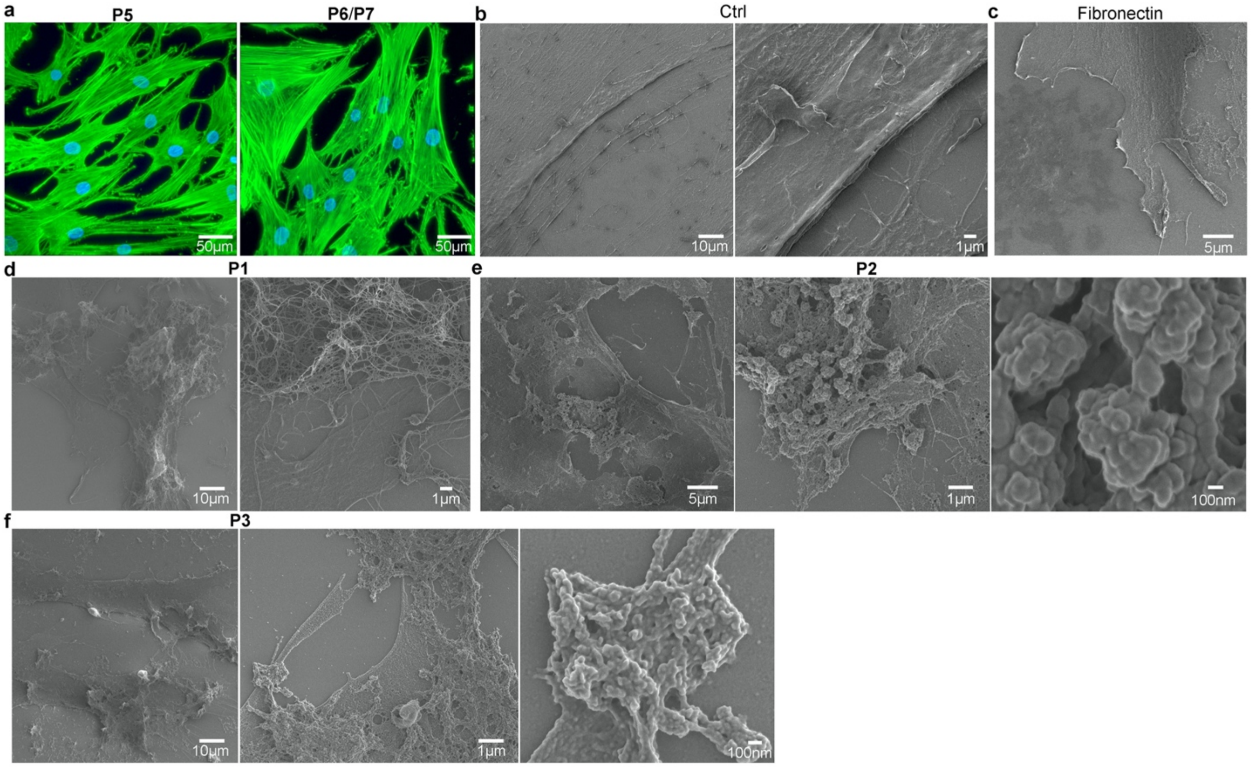

Figure S17. (**a**) Fluorescent images of hMSCs upon the treatment of **P5** and **P6/P7** at a total concentration of 200 μM in basal medium for 3 days co-stained with ActinGreen, DAPI and Congo Red. SEM images of hMSCs upon the treatment of 1xPBS (**b**), cultured on fibronectin coated surface (**c**), upon the treatment of **P1** (200 μM) (**d**), **P2** (200 μM) (**e**), and **P3** (200 μM) (**f**) in basal medium for 3 days.

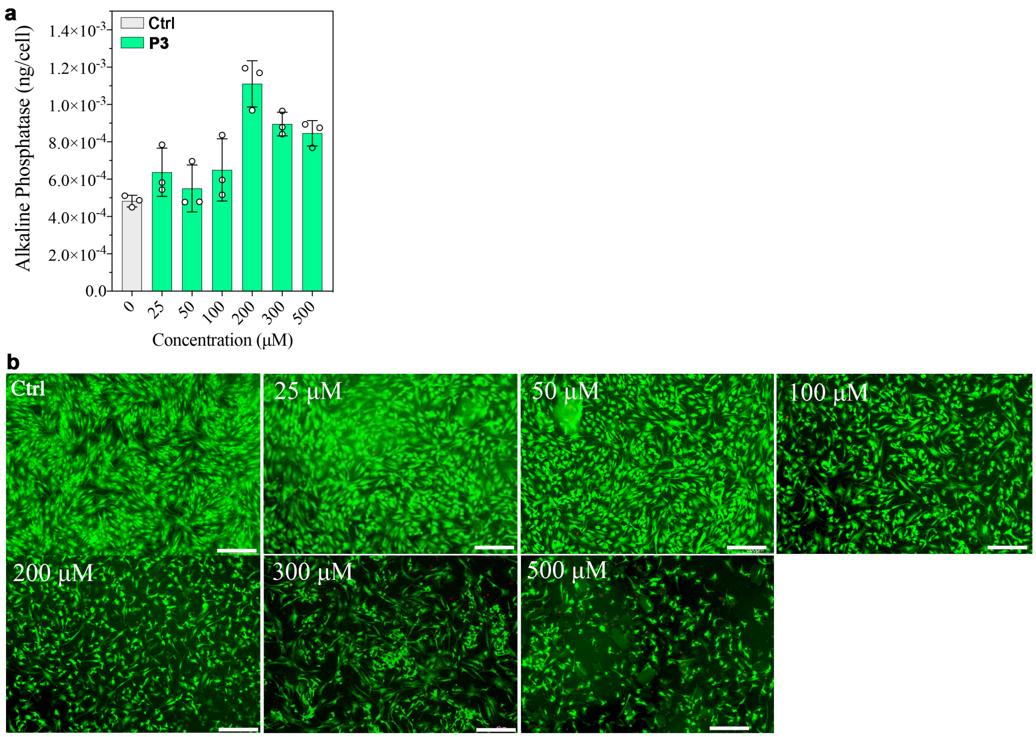

Figure S18. (**a**) ALP activity of hMSCs with and without the treatment of **P3** at various concentrations in osteogenic medium for 7 days. (**b**) Fluorescent images of hMSCs with and without the treatment of **P3** at various concentrations in basal medium for 3 days stained with live (green) and dead (red) cell indicator. The scale bars represent 200 μm.

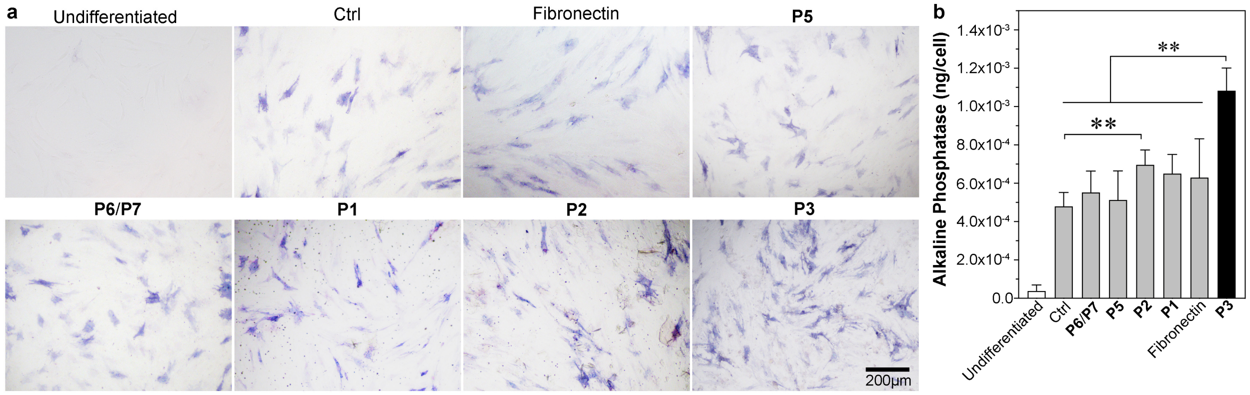

Figure S19. **(a)** ALP staining of hMSCs cultured in basal medium (undifferentiated); treated with 1xPBS (Ctrl), cultured on fibronectin coated surface (Fibronectin), treated with **P1-3**, **P5**, and **P6/P7** at a total concentration of 200 μM in osteogenic medium for 7 days. And the corelated quantitative results (mean ± SD, n = 3) **(b)**.

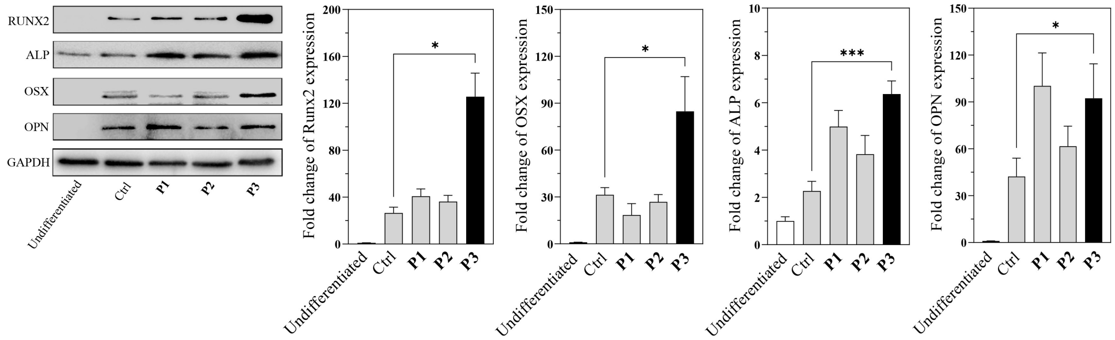

Figure S20. Immunoblotting analysis of RUNX2, OSX, ALP and OPN expression in hMSCs upon the treatment of **P1**, **P2** and **P3** at a concentration of 200 µM in osteogenic medium for 21 days. GAPDH serves as a loading control (mean ± SD, $n = 3$). * $p < 0.1$, ** $p < 0.01$, *** $p < 0.001$.

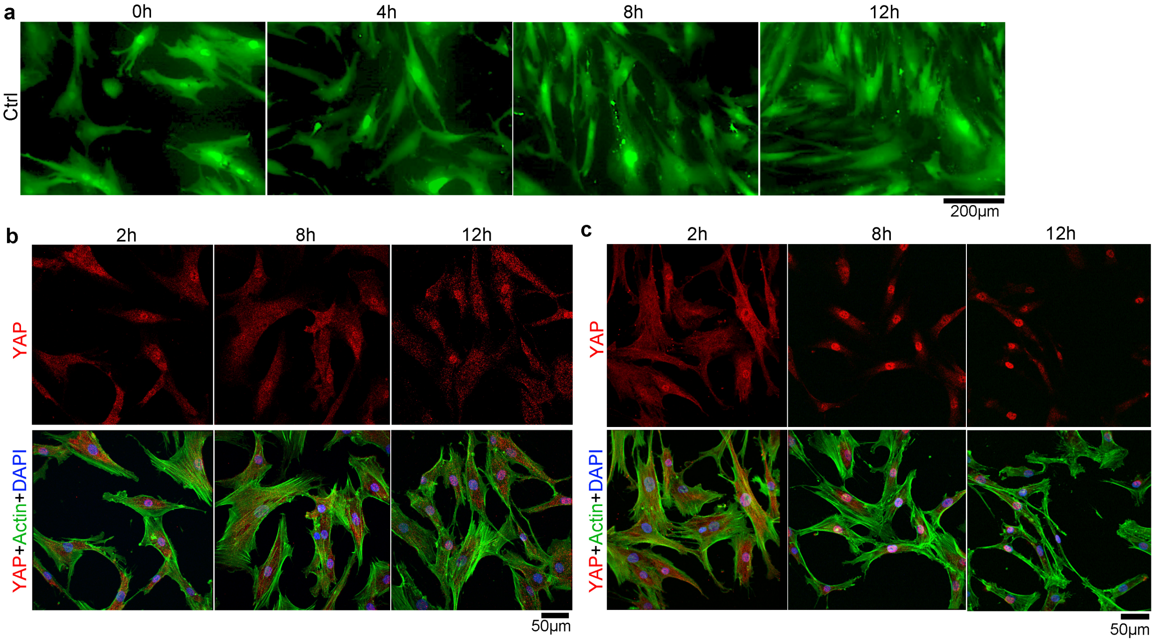

Figure S21. (**a**) Time-lapse images of hMSC-GFP treated with 1xPBS. Time-lapse IF imaging of YAP in hMSCs upon the treatment of 1xPBS (**b**), or **P3** (200 µM) in osteogenic medium (**c**) co-stained with ActinGreen and DAPI.

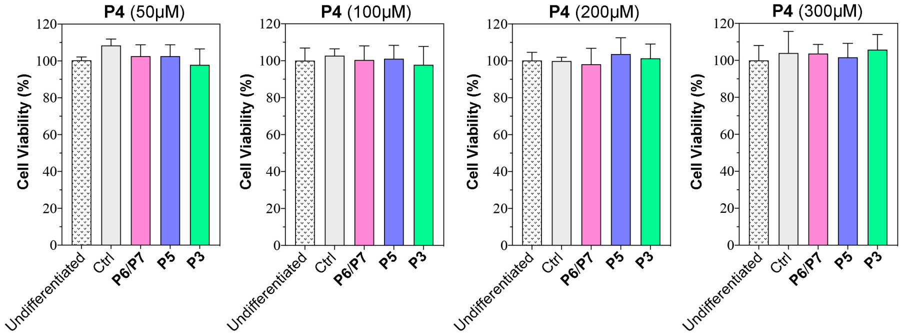

Figure S22. 6-day hMSC viability upon the treatment of peptides **P3**, **P5**, and mixture of **P6**/**P7** at a concentration of 200 µM for 3 days, followed with another 3-day treatment of **P4** at various concentrations in osteogenic medium (mean ± SD, *n* = 3). 'Undifferentiated' represents cell cultured in basal medium; 'Ctrl' represents cell cultured in osteogenic medium upon the treatment of 1xPBS buffer.

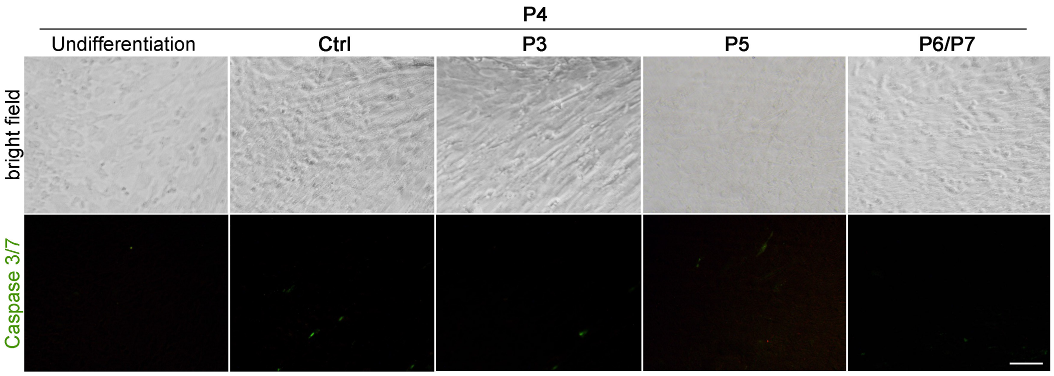

Figure S23. Bright field images and fluorescence images of hMSCs upon the treatment of peptides **P3**, **P5**, and mixture of **P6**/**P7** at a concentration of 200 µM for 3 days, followed with another 3-day treatment of **P4** at a concentration of 200 µM in osteogenic medium. 'Undifferentiated' represents cell cultured in basal medium; 'Ctrl' represents cell cultured in osteogenic medium upon the treatment of 1xPBS buffer. The scale bar represents 100 µm.

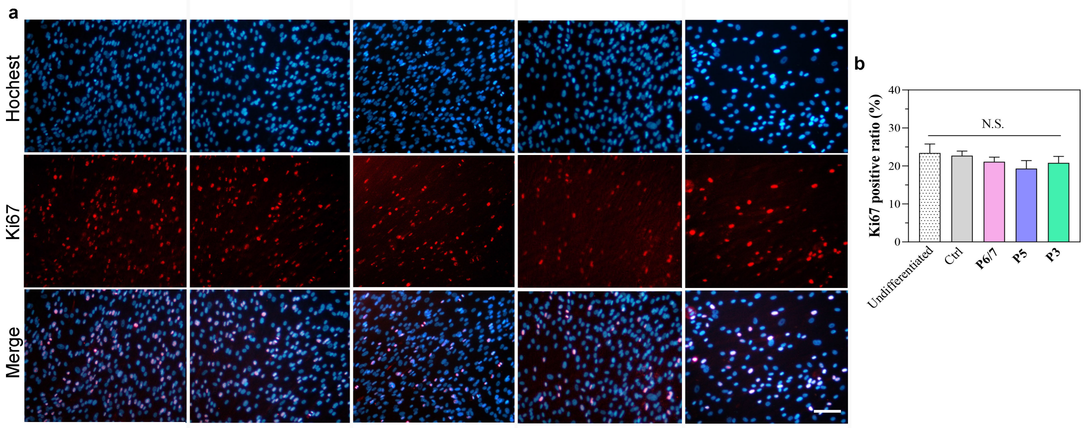

Figure S24. (a) Fluorescence images of hMSCs upon the treatment of peptides **P3**, **P5**, and mixture of **P6**/**P7** at a concentration of 200 µM for 3 days, followed with another 3-day treatment of **P4** at a concentration of 200 µM in osteogenic medium. 'Undifferentiated' represents cell cultured in basal medium; 'Ctrl' represents cell cultured in osteogenic medium upon the treatment of 1xPBS buffer. The scale bar represents 100 µm. **(b)** Quantification of Ki67 positive ratio in hMSCs upon the treatments of panel a (mean ± SD, *n* = 3).

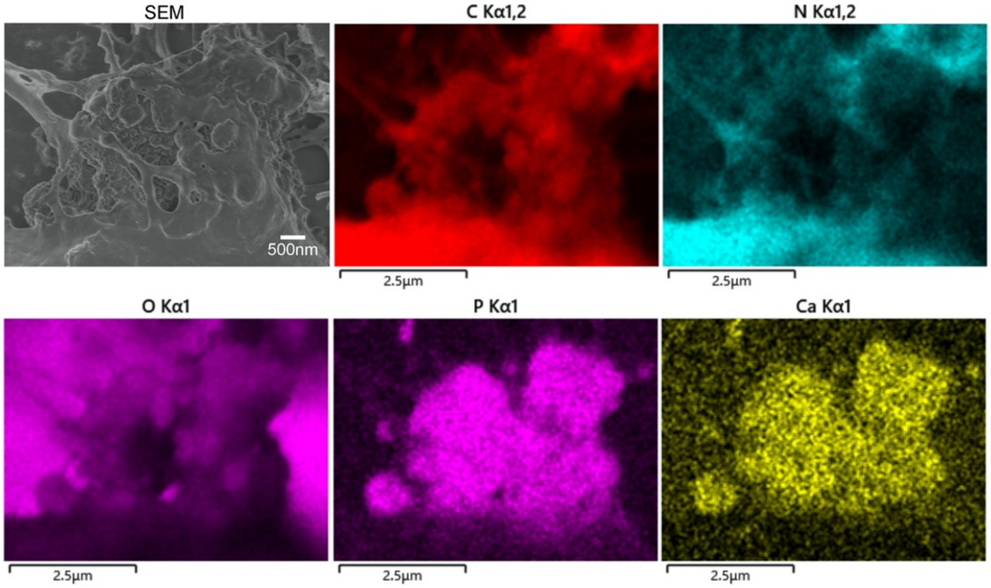

Figure S25. SEM image and the correlated EDS mapping of hMSC surface (Figure 5b area1) sequentially rerated by **P3** (200µM) and **P4** (200 µM) during osteogenesis.

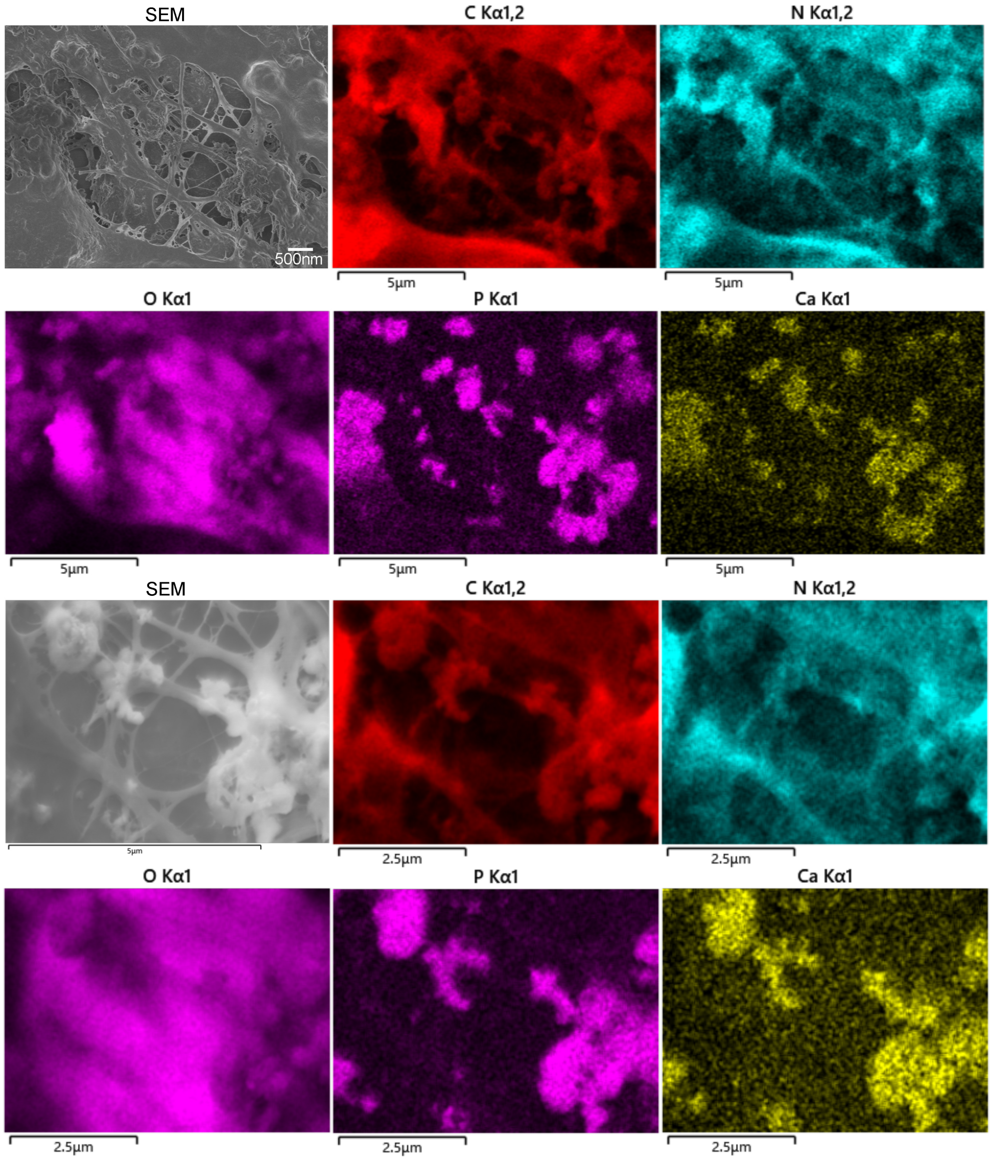

Figure S26. SEM image and the correlated EDS mapping of hMSC surface (Figure 5b area2) sequentially rerated by **P3** (200μM) and **P4** (200 μM) during osteogenesis.

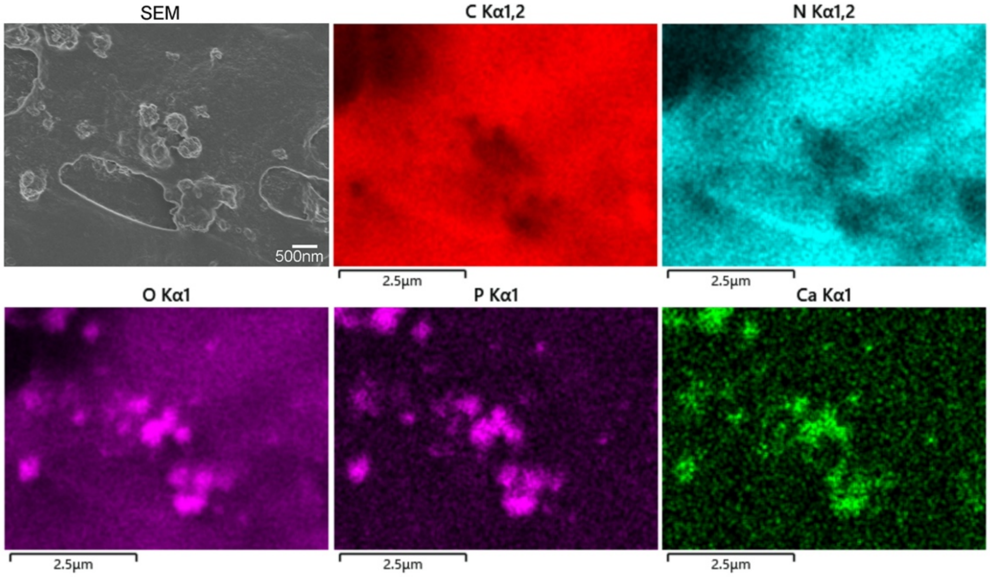

Figure S27. SEM image and the correlated EDS mapping of hMSC surface (Figure 5b area3) sequentially rerated by **P3** (200μM) and **P4** (200 μM) during osteogenesis.

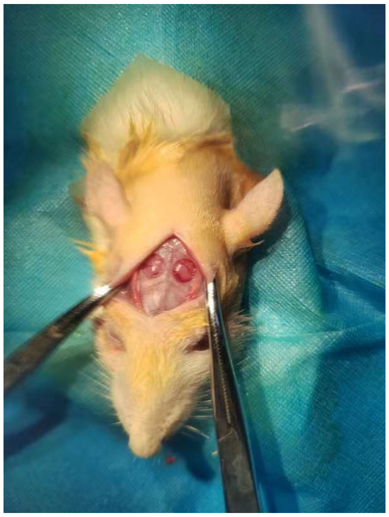

Figure S28. Photograph of cranial defects (8mm diameter) generated in a rat model.

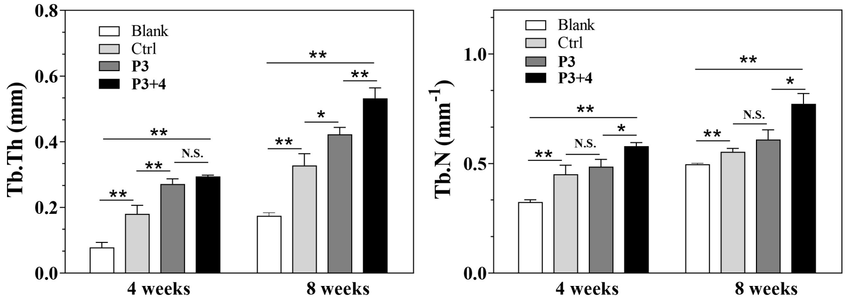

Figure S29. Trabecular thickness (Tb. Th) (left panel) and trabecular number (Tb. N) in calvarial defects by microCT after various implantations for 4 and 8 weeks.

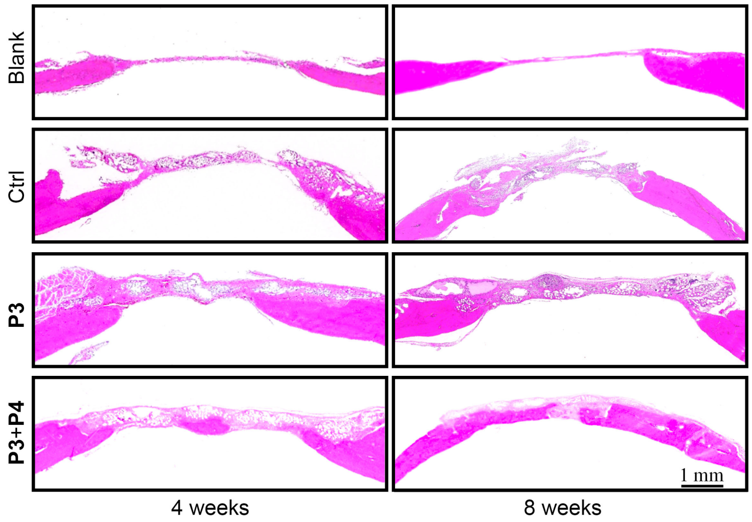

Figure S30. Hematoxylin and eosin (H&E) staining in calvarial defects after various implantations for 4 and 8 weeks.
